# Bioinspired Prolactin Pulse Release from Responsive Microneedles for Inhibiting Fatty Liver Formation

**DOI:** 10.1002/advs.202508364

**Published:** 2025-06-30

**Authors:** Hongli Yin, Wenjuan Tang, Danqing Huang, JingJing Gan, Pengzi Zhang, Yuanjin Zhao, Yan Bi

**Affiliations:** ^1^ Department of Endocrinology Endocrine and Metabolic Disease Medical Center Nanjing Drum Tower Hospital Affiliated Hospital of Medical School Nanjing University Nanjing 210008 China; ^2^ Branch of National Clinical Research Centre for Metabolic Diseases Nanjing 210008 China; ^3^ Department of Rheumatology and Immunology Nanjing Drum Tower Hospital Affiliated Hospital of Medical School Nanjing University Nanjing 210002 China; ^4^ State Key Laboratory of Bioelectronics School of Biological Science and Medical Engineering Southeast University Nanjing 210096 China

**Keywords:** drug delivery, hormone therapy, metabolic dysfunction, microneedle, prolactin, steatotic liver disease

## Abstract

Hormonal mechanisms of fatty liver formation require in‐depth exploration, and corresponding therapeutic strategies are urgently needed. Here, through serological testing of mice with fatty liver, it is found that the rhythms of prolactin secretion are disturbed and that the circulating prolactin level is reduced. Based on these findings, prolactin's biological effects on fatty liver are investigated, and biomimetic photothermal‐responsive core–shell microneedles with periodically prolactin releasing are proposed to inhibit lipid accumulation in liver. The microneedles are comprised of poly(lactic‐*co*‐glycolic acid) shells and prolactin‐loaded cores of photothermal black phosphorus, phase‐change gelatin, and carrageenan. Under periodically stimulation of near infrared (NIR), the prolactin in the microneedles can be released in a rhythmic manner for inhibiting the lipid accumulation in liver cells. Based on these features, it is demonstrated in mice with fatty liver that the bioinspired‐responsive microneedles can facilitate prolactin mitigating hepatic steatosis through its interaction with the hepatic prolactin receptor (PRLR) and by modulating the expression of fatty acid translocase (FAT/CD36). Thus, these photothermal‐responsive core–shell microneedles with prolactin pulse release hold significant promise for the treatment of fatty liver disease.

## Graphical abstract

Inspired by the prolactin secretion profile under normal physiological conditions, biomimetic prolactin‐releasing microneedles are designed. Under the control of near infrared (NIR), these responsive microneedles facilitate pulsed release of prolactin to inhibit fatty liver formation.

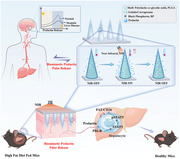

## 1 Introduction

The global prevalence of metabolic dysfunction‐associated steatotic liver disease (MASLD) is ≈38%, causing serious healthy burdens worldwide.^[^ ^1^, ^2^ ^]^ Currently, for the treatment of MASLD, endocrine hormones, including thyroid hormone, glucagon like peptide‐1 (GLP‐1), and fibroblast growth factor‐21 (FGF21), have attracted significant attentions. Various therapeutic strategies have been developed based on them. For example, as a kind of thyroid hormone receptor beta agonist, resmetirom has been proven to have the effect of reducing liver lipids.^[^ ^3^, ^4^ ^]^ Besides, GLP‐1 receptor agonist and FGF21 analogs have become the forefront research directions in the MASLD treatment.^[^ ^5^, ^6^ ^]^ Despite significant progress of endocrine hormones as therapeutic targets, the treatment results are not entirely satisfactory as the pathogenesis of MASLD is complex and in‐depth understanding of its mechanisms is still limited. Consequently, the development of effective, well‐tolerated, and safe therapeutic agents is imperative.

**Figure 1:**
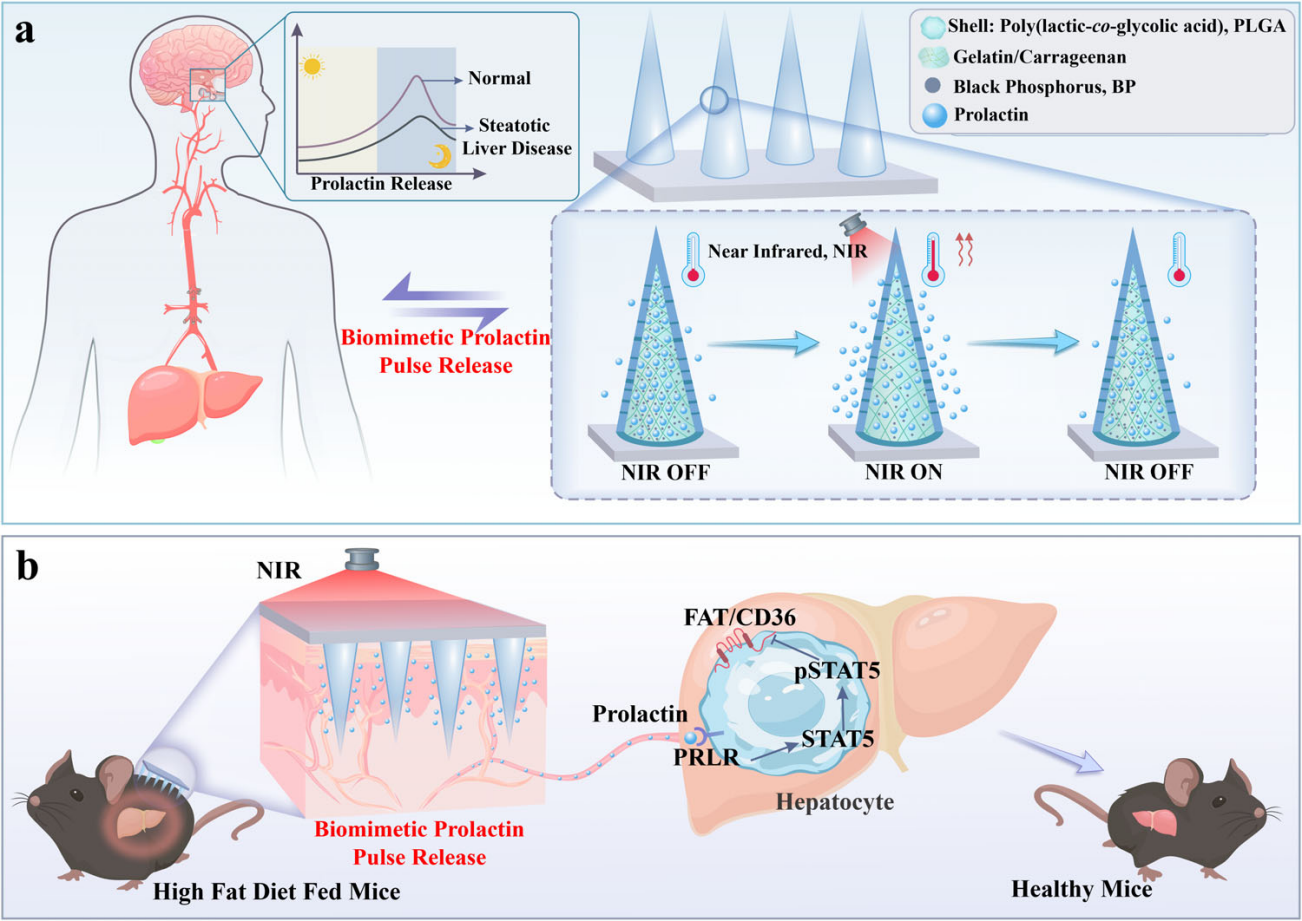
Schemes of the near‐infrared (NIR)‐responsive core–shell microneedle patch and its application for steatotic liver disease treatment. a) Design of the biomimetic prolactin releasing microneedles inspired by human prolactin release characteristics. b) NIR controlled prolactin pulse release for inhibiting fatty liver formation in MASLD mouse.

Here, by comparing the hormone secretion profile between healthy and MASLD mice, we found that the prolactin level was significantly decreased and the pulse secretion was weakened in MASLD mice. Based on this finding, we proposed a new biomimetic hormone delivery system to simulate the prolactin secretion profile under normal physiological conditions for inhibiting fatty liver formation, as schemed in **Figure** **1**. Previous studies have revealed that prolactin secreted by the pituitary gland adheres to circadian rhythms.^[^ ^7^, ^8^, ^9^ ^]^ Additionally, prolactin has a metabolic protective effect on maintaining liver lipid metabolism homeostasis in human.^[^ ^10^, ^11^, ^12^ ^]^ However, in fatty liver patients, circulating prolactin levels are reduced, and the profile of prolactin secretion undergoes alterations.^[^ ^13^, ^14^, ^15^, ^16^ ^]^ Although the effect of prolactin on fatty liver has been largely confirmed clinically,^[^ ^15^, ^17^ ^]^ prolactin's protective effect on lipid metabolism has not been deeply explored. In contrast, microneedles are popular drug carriers nowadays.^[^ ^18^, ^19^, ^20^, ^21^, ^22^ ^]^ Through penetrating the skin, microneedles can efficiently administrate drugs, not only reducing patient pain but also showing regulated drug release kinetics.^[^ ^23^, ^24^, ^25^, ^26^, ^27^ ^]^ Therefore, we conceived that integrating prolactin into intelligent microneedles can realize fatty liver treatment through pulsing prolactin release.

In this study, we developed a novel photothermal‐responsive core–shell microneedle to realize prolactin pulse release for inhibiting fatty liver formation. Through a repetitive mold replication method, core–shell structure microneedles with shell composing poly(lactic‐*co*‐glycolic acid) (PLGA), and prolactin‐loaded core composing photothermal black phosphorus (BP), phase‐change gelatin, and carrageenan were fabricated. The PLGA shell imparted the microneedles with adequate mechanical strength for skin penetration and facilitated drug release. By stimulating the microneedles with near infrared (NIR), BP in the core can absorb and converse NIR into thermal energy, bringing about reversible phase transition of gelatin/carrageenan and prolactin release. It was demonstrated that under periodically NIR irradiation, the prolactin can be released in a rhythmic manner for inhibiting the lipid accumulation in liver cells. Thus, the released prolactin in mice consuming a high‐fat diet (HFD) can mitigate hepatic steatosis through its interaction with the hepatic prolactin receptor (PRLR) and by modulating the expression of CD36, a pivotal fatty acid translocase (FAT) of free fatty acid (FFA) uptake. These results indicated that our photothermal‐responsive core–shell microneedles with prolactin pulse release can be a promising stratagem for the MASLD treatment.

## 2 Results and Discussion

### 2.1 Changes of Prolactin Secretion Level and Profile in MASLD Mice

Recently, the term MASLD has supplanted non‐alcoholic fatty liver disease (NAFLD).^[^ ^28^ ^]^ MASLD is a clinically heterogeneous disease characterized by excessive fat accumulation in hepatocytes and has a complex pathogenesis.^[^ ^28^, ^29^ ^]^ Emerging evidence has highlighted the significant relationship between MASLD and endocrine dysfunction, especially disruptions in pituitary hormones.^[^ ^17^, ^30^ ^]^ The pituitary gland, an integral component of the central nervous system, serves as a crucial endocrine gland that significantly modulates lipid metabolism.^[^ ^15^, ^17^, ^31^, ^32^ ^]^ Prolactin has been reported to function as a metabolic hormone, playing a crucial role in maintaining and promoting metabolic homeostasis.^[^ ^15^, ^17^, ^33^ ^]^ Extensive research has demonstrated prolactin's protective metabolic effect on human liver lipid metabolism homeostasis.^[^ ^10^, ^11^, ^12^ ^]^ Recent large‐cohort clinical researches have demonstrated that decreased serum prolactin concentrations are positively correlated with a heightened susceptibility to metabolic disorders, including obesity and MASLD.^[^ ^13^, ^14^, ^15^, ^16^ ^]^

**Figure 2:**
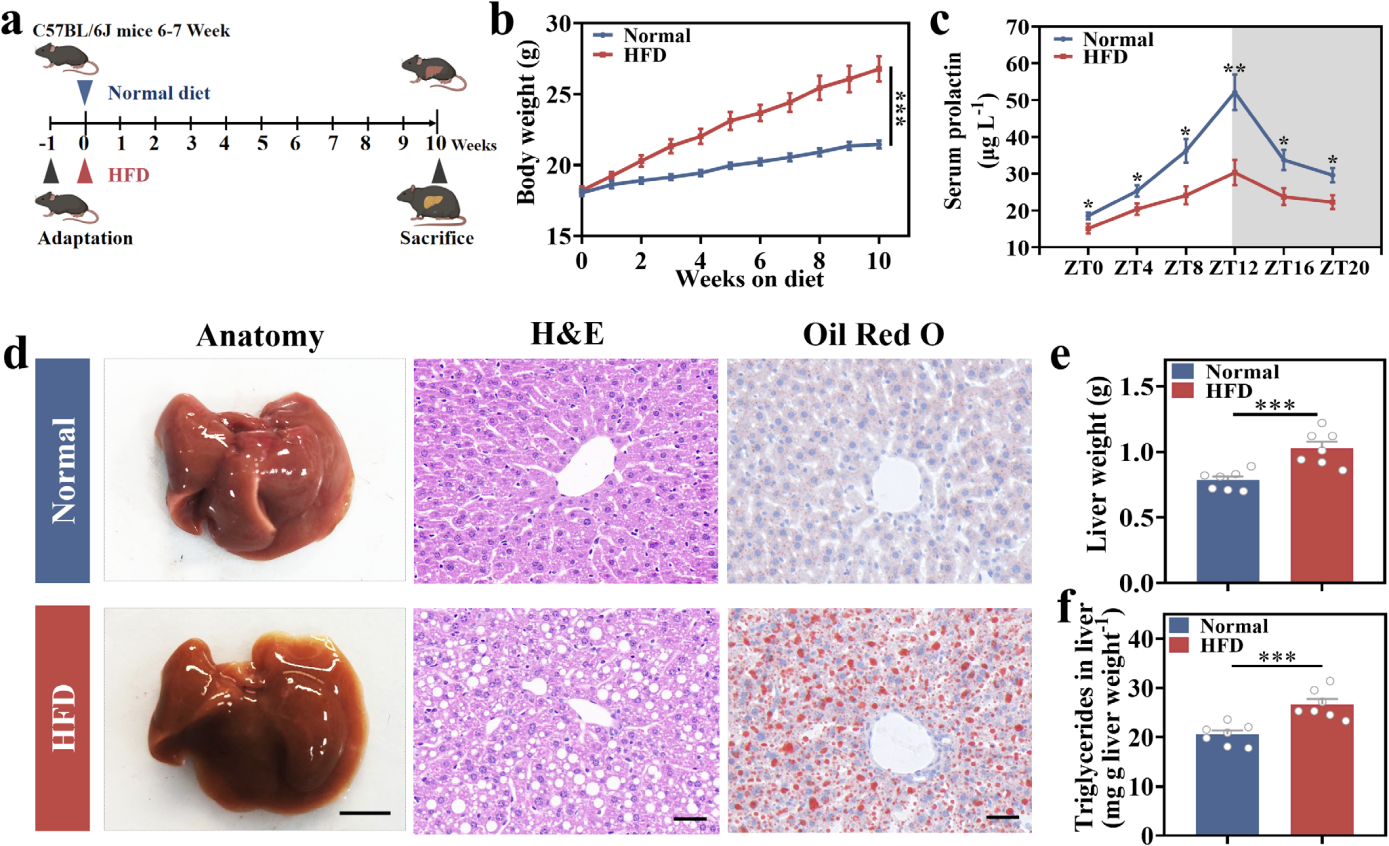
The analysis of serum prolactin levels in MASLD mice. a) Mouse modeling protocol. b) Weight changes of normal and HFD mice. c) Changes of serum prolactin levels between HFD and normal mice every 4 h within 1 day (ZT0 denotes the time at which light is on, while ZT12 signifies when light is off). d) The provided images include a macroscopic view of the mouse liver, with images featuring 0.5 cm scale bars, HE staining for cellular architecture and Oil Red O staining facilitated the visualization of lipid droplets within the liver tissue, with images featuring 40 µm scale bars. e) Liver weight and f) triglyceride levels of both normal and HFD mice. b,c,e,f) Mean ± standard error of the mean (SEM), *n* = 7.

To observe and quantitatively assess alterations in circulating prolactin concentrations in normal and MASLD mice, healthy 6–7 week female C57BL/6J mice were randomly allocated to two distinct groups. One acted as the normal group (normal diet), while the other was subjected to an HFD group (HFD diet) (**Figure** **2**a). Weekly weight changes of the mice were recorded (Figure 2b). After 10 weeks, compared with the normal group, the HFD group's body weight was significantly elevated. Furthermore, blood samples were gathered from the mice tail vein at zeitgeber time (ZT) 0 (8 a.m.), ZT4 (12 p.m.), ZT8 (4 p.m.), ZT12 (8 p.m.), ZT16 (12 a.m.), and ZT20 (4 a.m.) on the subsequent day to assess prolactin levels. These measurements revealed that the serum prolactin levels in the HFD group were markedly decreased than those in the normal group at corresponding time points, with a notable reduction in the peak of prolactin pulse release (Figure 2c). The histological examination, conducted using hematoxylin and eosin (HE) and Oil Red O staining, revealed significant hepatic steatosis in mice fed an HFD after 10 weeks (Figure 2d).^[^ ^17^, ^34^ ^]^ At the end of this duration, measurements revealed significantly elevated liver weights and triglyceride levels in the HFD group than the normal group (Figure 2e,f). Preliminary studies have shown reduced prolactin secretion in both patients with fatty liver and mice exhibiting hepatic steatosis.^[^ ^15^, ^17^ ^]^ Similarly, research has demonstrated that the peak pulse of prolactin release is diminished in overweight individuals compared to those of normal weight.^[^ ^35^ ^]^ Given that the secretion profile of endocrine hormones is crucial for their efficacy and, in some cases, determines the nature of their effects, it is essential to account for the level profile of hormones when designing drug delivery systems and treatment schedules involving the endocrine system.^[^ ^9^, ^12^ ^]^ Consequently, we propose integrating prolactin into smart microneedles to mitigate hepatic lipid accumulation through biomimetic prolactin release.

### 2.2 Preparation and Characterization of Gelatin/Carrageenan/BP Mixtures

Gelatin, a naturally biodegradable material derived from collagen, exhibits rapid and reversible phase transition characteristics in response to temperature changes.^[^ ^36^, ^37^, ^38^ ^]^ Doping carrageenan into gelatin solution can regulate the critical temperature of phase‐change materials.^[^ ^37^, ^39^ ^]^ To screen gelatin and carrageenan concentration ratio in microneedle core phase‐change materials, we observed that the gelatin phase transition temperature slightly increased (29.2–32 °C) with the increase of gelatin concentration (15–25%) (Figure S1a, Supporting Information). However, when carrageenan was doped in gelatin, the mixed hydrogel's phase transition temperature significantly increased (38–50 °C) with slightly increased carrageenan concentration (0.6–1.5%) (Figure S1b, Supporting Information). The phase transition temperature of a 20% gelatin doped with 0.65% carrageenan was around 39.3 °C (Figure S1c,d, Supporting Information). This temperature avoids uncontrolled melting at body temperature (36.5–37.5 °C) due to lower phase transition temperature. More importantly, the 39.3 °C phase transition temperature poses no risk of skin burns and protein drug denaturation due to higher phase transition temperature. Considering the mechanical strength and phase transition temperature of the hydrogel, we chose 20% gelatin doped with 0.65% carrageenan as the phase‐change material of the microneedle core in subsequent experiments.

With the purpose of investigating the NIR response and phase transition capability of microneedle core materials, we conducted experiments on photothermal conversion and phase transition processes during their NIR response. In this study, BP was chosen as a microneedle core photothermal conversion material due to its superior NIR absorption performance, photothermal conversion efficiency, satisfactory biocompatibility, and low toxicity.^[^ ^37^, ^40^, ^41^, ^42^ ^]^ A mixture of 20% gelatin and 0.65% carrageenan was prepared and was added to BP solution (0.2 mg mL^−1^). When the phase‐change hydrogel was exposed to NIR laser (808 nm), the BP in the combination hydrogel effectively absorbed light energy and transform it into heat. The results demonstrated that as the NIR power increased, the local temperature gradually rose over time (Figure S2a,b, Supporting Information). Notably, under a 1.5 W cm^−2^ NIR laser, the temperature could reach 39.3 °C within 2 min (Figure S2a,c, Supporting Information). Furthermore, comparing the temperature rise of the phase‐change core material under continuous irradiation with a 1.5 W cm^−2^ NIR laser with and without BP, it was observed that a significant temperature rise process occurred only in the presence of BP (Figure S2c,d, Supporting Information). This suggests that the phase‐change materials BP/gelatin/carrageenan can effectively respond to NIR irradiation.

### 2.3 Preparation of Responsive Microneedle Patch

**Figure 3:**
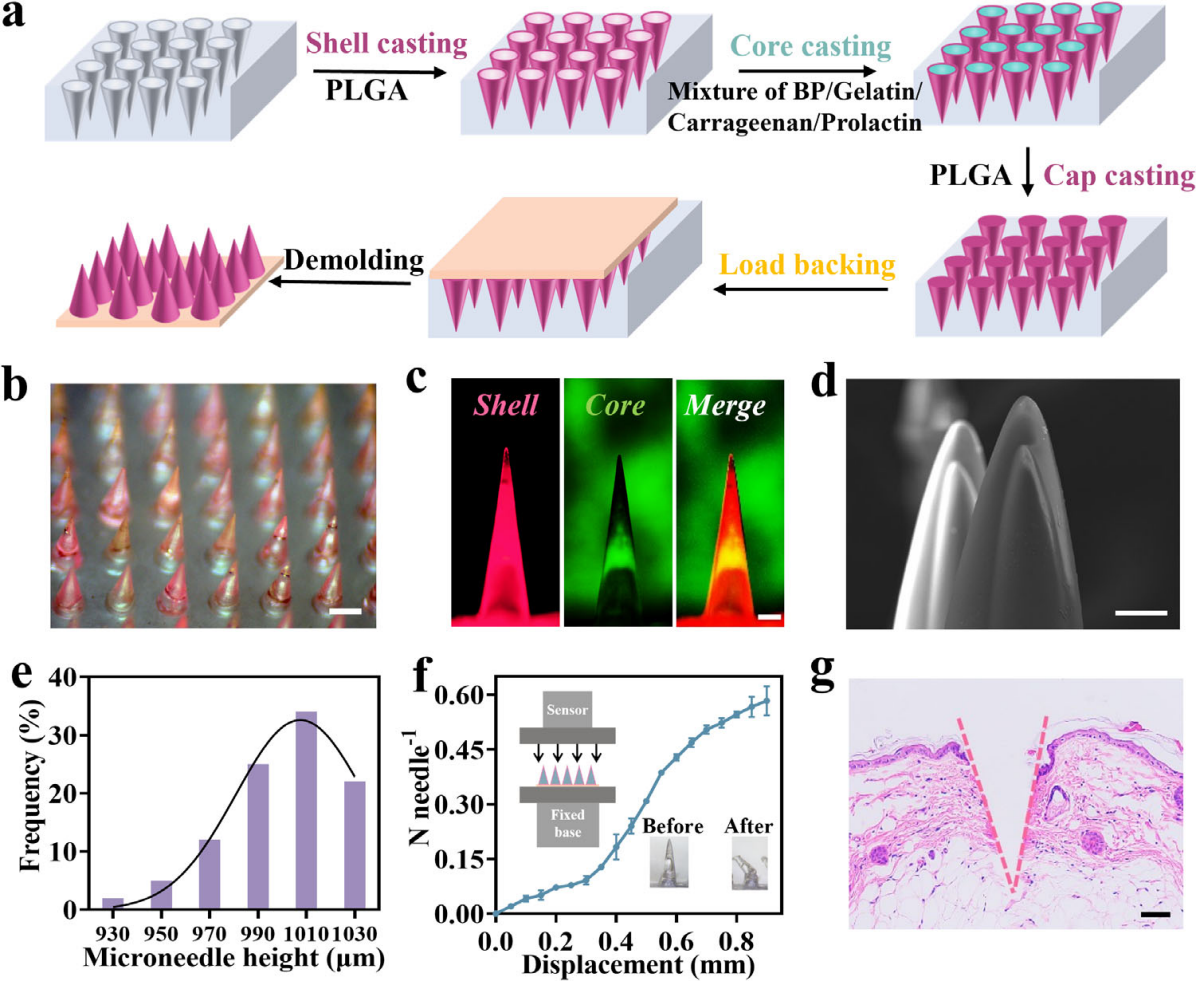
Preparation and characterization of microneedles. a) Manufacturing process diagram of photothermal‐responsive core–shell microneedles. b) Optical microscope image of photothermal‐responsive core–shell microneedles (scale bar = 450 µm). c) Fluorescence characterization of core–shell structure (scale bar = 200 µm). d) The core–shell microneedles’ scanning electron microscope  images (scale bar = 50 µm). e) Statistics pertaining to the uniformity of needle height. f) Compressive force of photothermal‐responsive core–shell microneedles (mean ± standard deviation (SD), *n* = 3). g) HE staining image of photothermal‐responsive core–shell microneedles penetrating the skin (scale bar = 100 µm).

PLGA has been chosen for the fabrication of microneedle shells due to its mechanical strength, exceptional biocompatibility,^[^ ^43^ ^]^ biodegradability,^[^ ^44^ ^]^ and superior film‐forming attributes.^[^ ^45^ ^]^ Polyvinyl acetate (PVA) serves as the backing layer for microneedles.^[^ ^46^ ^]^ In this research, we fabricated photothermal‐responsive core–shell microneedles using a mold method, with the specific procedural steps illustrated in **Figure** **3**a. Initially, a 10% PLGA acetone solution was introduced into the polydimethylsiloxane (PDMS) microneedle mold. This was followed by centrifugation and drying to generate a PLGA film on each microneedle cavity's inner surface. The thickness of the microneedle shell can be modulated by adjusting the number of PLGA film formations and the PLGA concentration. Subsequently, the optimized phase‐change core material was inserted into the microneedle shell while in a fluid state at 39.3 °C, and any excess phase‐change material was removed. Once the phase‐change material solidified, a 10% PLGA solution was added to the mold. This was then centrifuged and the solvent evaporated to form a cap layer atop the microneedles. Lastly, a 20% PVA solution was introduced into the mold to create a backing layer.

### 2.4 Characterization of Responsive Microneedles

The microneedle patch comprise a 10 × 10 cone‐shaped array, with ≈ 450 µm bottom diameter, and ≈1040 µm height. As demonstrated in Figure 3b and in Figure S3 (Supporting Information), we observed the fabricated core–shell microneedle array using an optical microscope and characterized its surface morphology with a scanning electron microscope. To visualize microneedles’ core–shell structure, we utilized a fluorescence microscope. The core of the microneedles, loaded with fluorescein isothiocyanate‐bovine serum albumin (FITC–BSA),^[^ ^47^ ^]^ exhibited green fluorescence, while the shell, labeled with Oil Red O, displayed red fluorescence (Figure 3c). Additionally, the presence of microneedle core–shell structures was confirmed under scanning electron microscope (Figure 3d). Then, we measured the height of the microneedles, over 80% of the microneedle heights fall within the range of 980–1040 µm (Figure 3e).

### 2.5 Mechanical Performance Evaluation of Responsive Microneedles

To assess the skin penetration capability of photothermal phase‐change core–shell microneedles, tensile machine was utilized to determine their mechanical robustness. The force measurement results for microneedles demonstrated that each injection needle could withstand a force of 0.58 ± 0.04 N (Figure 3f) prior to bending or breaking. This significantly surpasses the required force of 0.3 N per needle needed to penetrate the skin,^[^ ^48^ ^]^ ensuring that the microneedles’ patch effectively delivers the drug into the body. When the microneedles’ patch was applied to mice skin, HE staining indicated that the microneedles successfully penetrated the skin (Figure 3g).

### 2.6 Phase Transition Performance of Responsive Microneedles

**Figure 4:**
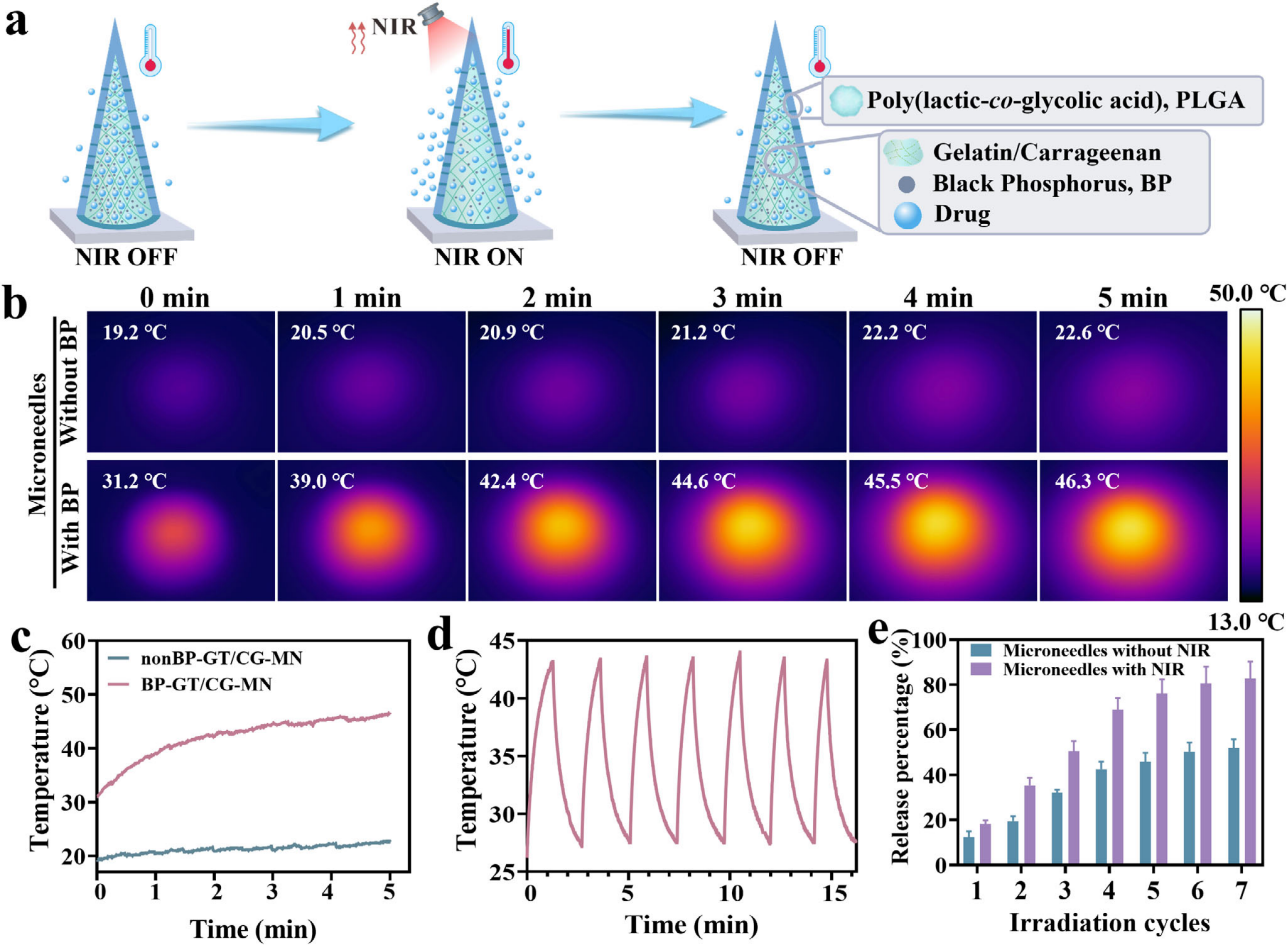
Evaluation of photothermal responsiveness and in vitro release characteristics of drug‐loaded microneedles. a) Schematic diagram of photothermal response of microneedles. b) In vitro photothermal response thermograms of microneedles without and with BP. c) In vitro photothermal response heating curves of microneedles without and with BP. d) The heating characteristics of photothermal‐responsive microneedles under seven NIR irradiation cycles. e) The in vitro drug release characteristics of photothermal‐responsive microneedles with and without NIR irradiation (mean ± SD, *n* = 3 per group).

The photothermal‐responsive core–shell microneedles’ drug release mechanism is illustrated in **Figure** **4**a. To further investigate the photothermal response of phase‐change materials incorporated into microneedles, we observed that those containing BP exhibited a heating process under NIR irradiation. In contrast, microneedles without BP did not display a notable heating process (Figure 4b,c). Additionally, the microneedles retained their photothermal response performance after seven cycles of NIR irradiation (Figure 4d).

### 2.7 Microneedle Shells’ Water Absorption and Degradation Characteristics

PLGA is a biodegradable polymer approved by the U.S. Food and Drug Administration (FDA), known for its ability to achieve sustained release of various drugs, including proteins, peptides, and small molecules, with good biocompatibility. The release mechanism of PLGA‐encapsulated drugs primarily involves diffusion through water‐filled pores and/or erosion of the polymer matrix.^[^ ^49^, ^50^ ^]^ The rate and extent of water absorption and polymer degradation can significantly influence drug release. Thus, this study examined the water absorption and degradation of PLGA microneedle shells. Results indicate that the PLGA microneedle shell rapidly absorbs water within 10 min of immersion in a liquid environment and reaches a relatively stable level of water absorption within 45 min (Figure S4a, Supporting Information). The degradation process of PLGA is slow, with a mass loss of ≈10% over 1 week (Figure S4b, Supporting Information). These findings suggest that the PLGA microneedle shell can quickly absorb water after administration, allowing drugs to diffuse through the water‐filled voids and be absorbed by the body. Additionally, the slow degradation process maintains the microneedle shape and prevents sudden large dose releases of loaded drugs, thereby ensuring stable efficacy.

### 2.8 Evaluation of Responsive Microneedles’ In Vitro Drug Release

To examine the drug loading and release capabilities of peptides and protein drugs in this core–shell microneedle system, FITC–BSA, a representative of macromolecular protein therapeutics,^[^ ^47^ ^]^ was incorporated into the core of the core–shell microneedle. To compare the drug release profiles of core–shell microneedles with and without photothermal phase transition characteristics, the microneedles were embedded in agar blocks and exposed to NIR light every 1 h. Microscopic images were taken to observe the release of FITC–BSA from the microneedles, and the results indicated that the drug release was faster and more complete after NIR irradiation (Figure S5, Supporting Information). The green fluorescence intensity of the microneedle core diminished progressively with each NIR irradiation cycle, which preliminarily demonstrated the drug release ability of microneedles. To further quantitatively analyze the drug release of microneedles with and without photothermal phase transition characteristics, the cumulative release amount was measured. The microneedles were placed in phosphate buffered saline (PBS) solution, and NIR light was applied every hour. The FITC–BSA content was quantitatively measured by fluorescence. Above results showed that the drug release was faster and more complete after NIR irradiation (Figure 4e).

### 2.9 Biocompatibility of Responsive Core–Shell Microneedles

Before conducting in vitro and in vivo efficacy evaluations of responsive microneedle patches, it is essential to examine the impact of microneedle materials on 3T3 cell viability to assess their biocompatibility and cytotoxicity. The findings indicate that the microneedle materials exert a negligible detrimental effect on the proliferation of 3T3 cells, indicating satisfactory cell compatibility and low cytotoxicity (Figure S6a,b, Supporting Information). The hemolysis assay was used to assess the responsive microneedles’ blood compatibility. The hemolysis rates for the microneedle extracts at varying concentrations were significantly below the 5% standard threshold,^[^ ^51^ ^]^ with negligible red blood cell rupture (Figure S6c, Supporting Information). Subsequently, after inserting the microneedles into the back skin of mice and then removing them, it was observed that the skin had largely returned to normal after 120 min, with no signs of inflammation, redness, or swelling at the insertion site (Figure S6d, Supporting Information). Additionally, observation of mouse skin following microneedle insertion revealed an almost 100% skin penetration efficiency (Figure S6d, Supporting Information).

### 2.10 Selection of FFA Modeling Concentration

To examine‌ the influences of varying concentrations of FFA on normal mouse liver cells, AML‐12 cell viability was assessed in response to FFA concentrations of 100, 200, 300, 400, and 500 µm. The Calcein/popidium iodide (PI) assay and CCK‐8 method (Figure S7, Supporting Information) indicated that a concentration of 200 µm FFA did not impact cell viability.

### 2.11 Determination of Prolactin Administration Concentration In Vitro

Traditional studies have established that the normal physiological range for prolactin levels is 1–25 µg L^−1^.^[^ ^10^, ^12^ ^]^ Therefore, this study investigates the effects of prolactin at concentrations of 1, 10, 20, 50, 100, and 500 µg L^−1^ on AML‐12 cell viability.^[^ ^15^ ^]^ The results indicated that the specified concentration range did not impact AML‐12 cell viability (Figure S8a,b, Supporting Information). The improvement effect of prolactin at the above concentrations on lipid accumulation in AML‐12 cells caused by FFA modeling was further evaluated. The quantitative detection of triglyceride levels revealed that prolactin reduces cellular triglyceride levels in a dose‐dependent manner with effective concentrations ranging from 1 to 100 µg L^−1^ (Figure S8c, Supporting Information). However, no further reduction in triglyceride levels was observed when prolactin concentration increased from 100 to 500 µg L^−1^. Consistent with these findings, Oil Red O and boron‐dipyrromethene (BODIPY) staining in AML‐12 cells showed that prolactin notably reduces lipid accumulation in a dose‐dependent manner, with effective concentrations ranging from 1 to 100 µg L^−1^ (Figure S8d, Supporting Information), with no additional decrease observed at concentrations ranging from 100 to 500 µg L^−1^.

### 2.12 In Vitro Efficacy Evaluation of Prolactin‐Loaded Responsive Microneedles

**Figure 5:**
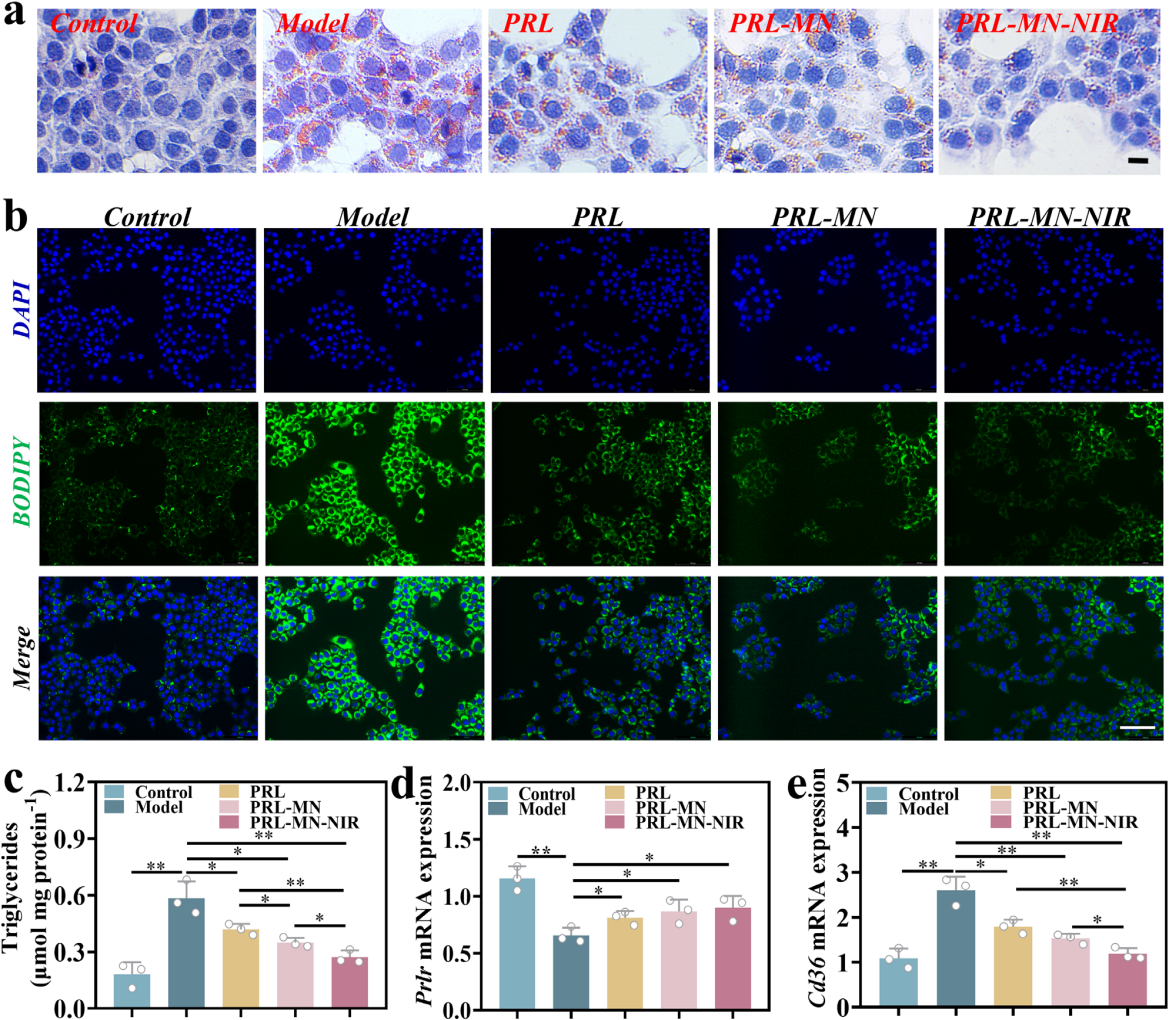
In vitro efficacy evaluation and mechanism of action of prolactin‐loaded responsive microneedles. a) Oil Red O staining, with images featuring a 20 µm scale bar and b) BODIPY staining, with a scale bar representing 100 µm were employed to evaluate the lipid‐lowering efficacy of each treatment group. c) Triglyceride levels of each group. d) The *Prlr* and e) *Cd36* mRNA expression levels of each group. c– e) Mean ± SD, *n* = 3.

To verify that the native structure of prolactin remained intact following patch fabrication and subsequent release, the secondary structure of the treated prolactin was analyzed using circular dichroism (CD) spectropolarimetry.^[^ ^52^ ^]^ The CD spectra of the treated prolactin exhibited two characteristic bands at 208 and 222 nm, indicative of alpha‐helical content, closely matching those observed for freshly prepared prolactin (Figure S9, Supporting Information). These results indicated that the native structure of prolactin can remain intact following patch fabrication and its subsequent release. To further evaluate the activity of prolactin‐loaded microneedles, a comparative study was conducted on AML‐12 cells modeled by FFA. The methods included direct administration of prolactin (PRL group), administration of prolactin‐loaded microneedles (PRL‐MN group), and administration of prolactin‐loaded microneedles combined with NIR irradiation (PRL‐MN‐NIR group). The results from AML‐12 cell Oil Red O staining (**Figure** **5**a), BODIPY staining (Figure 5b), and AML‐12 cell triglycerides level measurement (Figure 5c) indicate that responsive microneedle‐loaded prolactin combined with NIR irradiation can effectively reduce liver cell lipid accumulation. It is speculated that prolactin directly added to the culture medium can be quickly metabolized^[^ ^53^ ^]^ compared to the prolactin released by microneedles, which can slowly and stably exert its effect. After NIR irradiation, the microneedles loaded with prolactin can simulate the pulse release of prolactin under physiological conditions, with a more complete release of prolactin.

### 2.13 Exploring the Action Mechanism of Prolactin‐Loaded Microneedles In Vitro

In our previous study,^[^ ^15^ ^]^ we have investigated the action of prolactin/PRLR in hepatic lipid metabolism. The CD36 molecule is a crucial downstream target of class I cytokine receptors, such as PRLR, and is essential for hepatic FFA uptake. Liver tissue from patients with MASLD exhibited a marked reduction in *Prlr* gene expression and a concomitant elevation in *Cd3*6 gene levels relative to non‐MASLD controls. However, no correlations were observed between *Prlr* and genes involved in fatty acid oxidation (*Srebp1c* and *Acc1*) or de novo lipogenesis (*Pparα* and *Cpt1a*).^[^ ^15^ ^]^ In this study, we further investigate the mechanism by which prolactin reduces lipid accumulation in AML‐12 mouse normal liver cells. The findings revealed that prolactin administration significantly restored the reduced *Prlr* gene levels (Figure 5d) and suppressed the elevated *Cd36* gene levels induced by FFA modeling (Figure 5e). However, it had no impact on the gene levels of *Srebp1c*, *Acc1*, *Pparα*, *and Cpt1a* (Figure S10, Supporting Information). These findings are consistent with our previous studies on human liver tissue.^[^ ^15^ ^]^

### 2.14 In Vivo Efficacy Evaluation of Prolactin‐Loaded Responsive Microneedles

**Figure 6:**
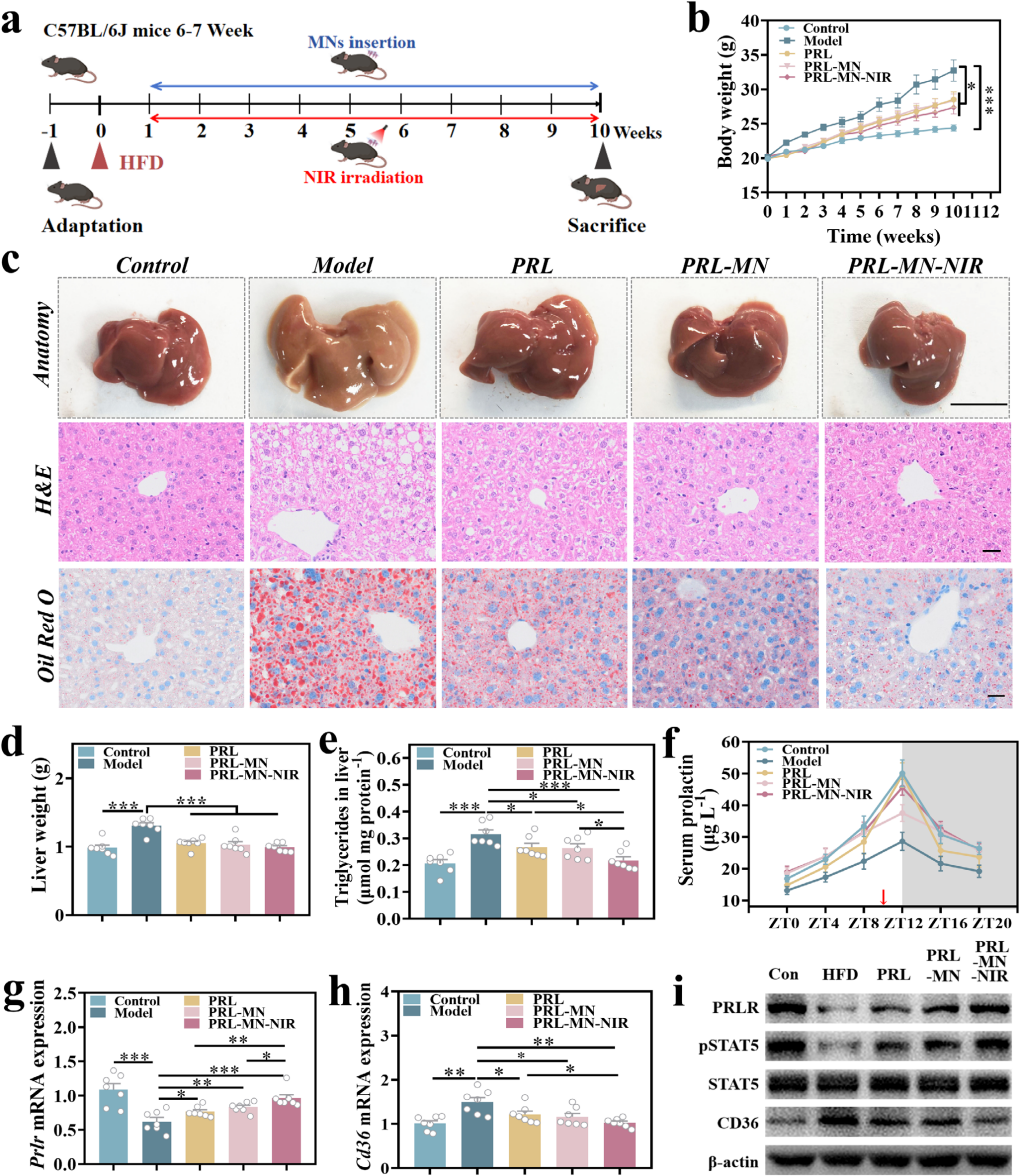
In vivo efficacy and mechanism of prolactin‐loaded responsive microneedles. a) Intervention plan for prolactin‐loaded responsive microneedle patch to inhibit liver steatosis in HFD‐induced mouse models. b) Weight changes observed in each group. c) Liver anatomy and histological sections stained with HE and Oil Red O from mouse liver samples, with images featuring 1 cm, 25 µm, and 25 µm scale bars, respectively. d) The liver weights and e) liver triglyceride levels of each group. f) The day before the mouse was euthanized, and the serum prolactin levels of each group mice changed every 4 h within 1 day (the red arrow indicates the time of administration). g) Determination of mice liver tissues *Prlr* and h) *Cd36* mRNA expression levels in each group. i) Protein levels of PRLR, pSTAT5, STAT5, and CD36 after interventions. b,d–h) Mean ± SEM, *n* = 7.

Initially, we inserted prolactin‐loaded responsive microneedles into the back of mice followed by NIR irradiation; it was found that the temperature could be raised to 39.3 °C within 53 s (Figure S11, Supporting Information). This suggests that microneedles may undergo a warming process in mice and accelerate the release of prolactin. Mice were randomly assigned to one of five distinct groups: the first group was given a normal diet (control group), while the second group received an HFD (model group), the third group received prolactin subcutaneously (PRL group), the fourth group received prolactin through microneedle administration (PRL‐MN group), and the fifth group received prolactin‐loaded microneedle combined with NIR irradiation (PRL‐MN‐NIR group) (**Figure** **6**a). Following 10 weeks of HFD administration, there was a notable rise in weight of mice than those on a normal diet (Figure 6b). The administration of prolactin significantly reduced the body weight of mice, with the combination of prolactin microneedles and NIR demonstrating the most effective weight reduction. After a 10 week modeling period, the mice were humanely euthanized, and their liver tissue was gathered. HE and Oil Red O staining of liver tissue section suggest that prolactin administration can significantly reduce mouse liver steatosis (Figure 6c). The above results showed that HFD modeling for 10 weeks significantly increased mouse liver weight and liver triglyceride levels, while prolactin administration could significantly reduce them (Figure 6d,e). What is more, the prolactin combined with NIR administration group showed the most significant efficacy. Blood samples were gathered from the mice tail vein at ZT0, ZT4, ZT8, ZT12, ZT16, and ZT20 on the day before euthanizing the mice. The results showed that both the prolactin subcutaneous injection group and the prolactin microneedle combined with NIR irradiation group significantly increased prolactin levels at ZT12 (8 p.m.). The prolactin microneedle combined with the NIR irradiation group could also increase prolactin levels at other time points in the blood (Figure 6f; Table S1, Supporting Information). In this study, prolactin is a protein‐based hormone that is easily degraded and inactivated by oral or intravenous administration. Therefore, the subcutaneous administration of the protein‐based hormone became the primary option. The subcutaneous region has relatively few capillaries and acts as a natural reservoir for the protein‐based hormone. This allows for the gradual release of the protein‐based hormone into the bloodstream, thereby extending its therapeutic effects over a longer period. Compared with intramuscular injection, subcutaneous injection can achieve a higher blood concentration and has a longer duration of action.^[^ ^54^ ^]^ Loading prolactin into responsive microneedles not only reduces patient pain but also demonstrates regulated drug release kinetics. Without NIR stimulation, prolactin‐loaded responsive microneedles can achieve slow and sustained release of prolactin at a relatively low level. Upon with NIR stimulation, the effective combination of fixed point high‐dose and low‐level sustained releases of prolactin is achieved, thereby better simulating the physiological secretion profile of prolactin.

### 2.15 Exploring the Action Mechanism of Prolactin‐Loaded Microneedles In Vivo

In our previous study,^[^ ^15^ ^]^ the prolactin/PRLR's action mechanism in vitro on lipid metabolism has been studied. In FFA‐induced HepG2 cells, prolactin considerably declined CD36 protein expression and promoted the phosphorylation of its critical upstream regulator, STAT5. Likewise, the overexpression of PRLR considerably declined the CD36 gene and protein expression, and promoted STAT5 phosphorylation. In contrast, the *Srebp1c*, *Acc1*, *Pparα*, and *Cpt1a* gene levels were not considerably affected. To delve deeper into the mechanism by which prolactin mitigates lipid accumulation in MASLD mice, quantitative real‐time polymerase chain reaction (qRT‐PCR) was utilized to quantify the transcriptional levels of *Prlr*, *Cd36*, *Srebp1c*, *Acc1*, *Pparα*, and *Cpt1a* genes in liver tissues from various treatment groups. These findings revealed that prolactin administration notably counteracted the decline in *Prlr* gene levels (Figure 6g) and suppressed the elevation in *Cd36* gene levels of MASLD mice liver (Figure 6h). However, prolactin administration exerted no influence on the gene levels of *Srebp1c*, *Acc1*, *Pparα*, and *Cpt1a* (Figure S12, Supporting Information). Western blot (WB) analyses of protein expression levels of PRLR, CD36, p‐STAT‐5, and STAT‐5 in the liver tissues across treatment groups indicated that prolactin administration significantly reverted the reduction in PRLR protein expression levels, curbed the upregulation of CD36 protein expression triggered by HFD modeling, and increased the phosphorylation of STAT‐5 protein (Figure 6i; Figure S13, Supporting Information).^[^ ^15^ ^]^ Tissue samples from the skin, heart, spleen, lungs, and kidneys of the mice were subjected to HE staining to scrutinize pathological alterations and ascertain the safety profile of the administration method. The observations confirmed that microneedle administration is devoid of adverse effects, does not inflict damage upon various organs, and does not cause inflammation of the skin at the site of administration (Figure S14, Supporting Information). In summary, the NIR‐responsive core–shell microneedle developed in this study is an effective and safe bionic hormone delivery strategy. This innovation not only offers a promising reference for the prevention and treatment of MASLD but also holds significant application potential in addressing hormone deficiency.

## 3 Conclusions

In this study, we designed biomimetic photothermal‐responsive core–shell microneedles with periodically prolactin releasing to inhibit lipid accumulation in liver. The microneedles were composed of PLGA shells and prolactin‐loaded cores of photothermal BP, phase‐change gelatin, and carrageenan. BP can absorb NIR, subsequently converting this energy into heat. This generated heat facilitates the heating of the phase‐change material, a gelatin/carrageenan composite, embedded within the microneedle's core. Upon reaching its designated phase transition temperature, the gelatin/carrageenan material undergoes a transformation from a solid to a liquid state, which can accelerate the release of the loaded drug. The phase transition temperature of the microneedle core phase‐change material is 39.3 °C, which prevents uncontrolled melting at body temperature and avoids excessive temperatures that could burn the skin. The PLGA shell provides the responsive microneedles with sufficient mechanical strength to penetrate the skin. After prolactin‐loaded microneedles administration, the PLGA microneedle shell rapidly absorbs water to form absorption pores, facilitating timely prolactin release. The slow degradation of the PLGA shell prevents sudden prolactin release and ensures controlled, sustained release of the encapsulated prolactin. Combined with NIR stimulation, prolactin‐loaded responsive microneedles can simulate the physiological secretion profile of prolactin and achieve controlled release.

The specific pharmacological mechanism of prolactin in MASLD treatment can be summarized as follows. A single prolactin molecule binds two PRLRs, thereby inducing their dimerization. Dimerized PRLRs exert their biological function through cellular kinases, including phosphoinositide 3‐kinase (PI3K)/protein kinase B (PKB, also known as AKT), STAT5, and Ras/mitogen‐activated protein kinase (MAPK).^[^ ^12^, ^15^ ^]^ Among these three signaling pathways, the activation of STAT5 ameliorates hepatic steatosis in HFD mice by suppressing CD36 expression.^[^ ^55^, ^56^ ^]^ Furthermore, we observed that phosphorylated STAT5 was increased after prolactin intervention or PRLR overexpression in HepG2 cells, but not the PI3K/AKT signaling pathway.^[^ ^15^ ^]^ Moreover, our previous studies have revealed that decreased serum prolactin levels under circadian misalignment conditions inhibit the activation of hepatic MAPK/Cyclin D1 signaling pathway, leading to the upregulation of lipogenic enzymes, such as fatty acid synthase and acetyl‐coa carboxylase, and subsequently results in hepatic steatosis.^[^ ^57^ ^]^ Collectively, prolactin primarily influences downstream targets of the PRLR, including STAT5 and Ras/MAPK pathways, thereby modulating genes associated with liver lipid synthesis and absorption, and then alleviates hepatic lipid accumulation. In this study, the pharmacological mechanisms of prolactin in vitro and in vivo are consistent with our previous studies.^[^ ^15^ ^]^ The findings of this research demonstrate that we have developed a safe and effective bionic delivery strategy for hormones. Specifically, the bionic delivery of prolactin has been shown to effectively inhibit lipid accumulation in the liver, thereby offering a novel approach for the prevention and treatment of MASLD.

MASLD is a clinically heterogeneous disease characterized by excessive fat deposition in hepatocytes.^[^ ^28^, ^29^ ^]^ Currently, clinical treatment options for MASLD are very limited. Previous studies, along with our research group's findings,^[^ ^15^, ^33^ ^]^ have demonstrated that prolactin secreted by the pituitary gland plays a crucial metabolic protective role in maintaining lipid metabolism homeostasis in the liver. Low levels of prolactin are identified as a risk factor for the onset and progression of MASLD.^[^ ^13^, ^14^, ^15^, ^16^ ^]^ Free fatty acids contribute to the development of fatty liver by suppressing prolactin levels.^[^ ^17^ ^]^ High‐fat diets and social jet lag can disrupt prolactin levels and rhythms, and restoring prolactin levels can help treat fatty liver.^[^ ^57^ ^]^ The findings indicate that normal prolactin levels and rhythms are essential for metabolic health.^[^ ^11^, ^58^ ^]^ In this study, we explored and proposed the use of bionic prolactin supplementation to inhibit the formation of fatty liver. Prolactin, a metabolic hormone, has increasingly garnered attention for its role in metabolic regulation.

In view of hormone deficiency, long‐term exogenous hormone supplementation is the main therapeutic strategy. To minimize the frequency of administration, the researchers mainly focused on the development of long‐acting hormones.^[^ ^26^, ^59^ ^]^ Other products with bionic delivery functions are currently being developed, primarily focusing on the treatment of diabetes.^[^ ^60^ ^]^ Presently, the application of bionic delivery for other hormones remains very limited. Prolactin, like most endocrine hormones, exerts its physiological effects through its secretion profile. This study investigated the biomimetic delivery mode of prolactin, which provided a useful reference for the biomimetic delivery of hormones.

In addition to the circadian rhythm of hormone secretion, there are small pulses released at various points in time. Furthermore, the secretion of prolactin is influenced by emotions, stress, etc.^[^ ^53^ ^]^ In this study, it was not possible to adjust hormone secretion in response to these influencing factors. Moreover, patients with hormone deficiencies require long‐term exogenous hormone supplementation, which poses significant inconvenience and places a substantial economic burden on their lives. In future studies, we aim to integrate advanced technical methodologies to develop new treatment strategy capable of releasing prolactin or other pituitary hormones to meet physiological needs. This approach holds promise for advancing personalized treatment strategies for hormone deficiencies and metabolic diseases such as MASLD.

## 4 Experimental Section

### Materials

Gelatin and PVA were purchased from Sigma–Aldrich. Carrageenan was bought from Shanghai Yuanye. BP was purchased from Nanjing XFNANO. PLGA (average *M*_w_ ≈ 4.8 W) was bought from Nanjing Geology. Recombinant murine prolactin (>98%, 197 amino acids, *M*_w_ ≈ 22.4 kDa) was purchased from Shanghai Prime Gene. FITC–BSA was procured from Bovogen. Dulbecco's modified eagle medium (DMEM) and fetal bovine serum (FBS) were purchased from USA Gibco. CCK‐8 and cell viability/cytotoxicity assay kit were obtained from Shanghai Beyotime. Prolactin enzyme linked immunosorbent assay (ELISA) kits were bought from Nanjing Fcmacs. The primers (Table S2, Supporting Information) were synthesized by Shanghai Generay Biotechnology. Anti‐PRLR (ab170935) and anti‐CD36 (ab252922) were provided by Abcam. Anti‐Stat5 (D2O6Y) and anti‐phospho‐Stat5 (D47E7) were provided by Cell Signaling Technology. The experimental water was deionized water.

### Cell Lines and Animals

NIH 3T3 and AML‐12 cells were sourced from the Cell Bank of Chinese Academy of Sciences. The cell culture process employed DMEM added with 10% FBS and 1% bispecific antibody. Healthy 6–7 week female C57BL/6J mice were obtained from Jiangsu Huachuang Sino Pharma Tech Co., Ltd. All animal experiments were conducted humanely and complied with the ethical guidelines established by the Institutional Animal Care and Use Committee of the Affiliated Drum Tower Hospital (Approval Number: 2024AE01027).

### Measurement of Prolactin Levels in Normal and MASLD Mice

The mice were haphazardly‌ assigned to two groups. The model group was administered an HFD comprising 60% fat for a duration of 10 weeks. Conversely, age‐matched control mice were accommodated a standard diet. Blood samples were gathered from the tail vein of mice at ZT0, ZT4, ZT8, ZT12, ZT16, and ZT20 on the subsequent day, prior to sacrificing the mice at the end of the 10th week of modeling to measure prolactin levels. The levels of prolactin were determined by prolactin ELISA kits. Upon sacrificing the mice, liver tissue was extracted to ascertain the weight and triglyceride levels. HE and Oil Red O staining were employed to observe the mouse liver steatosis.

### NIR Responsiveness Determination of BP/Gelatin/Carrageenan

To study the phase transition temperature, gelatin solutions of varying concentrations (15, 20, and 25 wt%) were prepared and transformed into firm state. The solid gelatin gel was heated, and subsequently the critical temperature at the onset of melting was recorded. Solutions with varying carrageenan concentrations (0.6, 0.65, 0.7, 0.8, 0.9, 1.2, and 1.5 wt%) were prepared, and the procedure was replicated. To study NIR responsiveness, a focused NIR laser with powers of 1.0, 1.25, and 1.5 W cm^−2^ was used to vertically irradiate BP/gelatin/carrageenan (BP 0.2 mg mL^−1^). Concurrently, temperature variations were documented using a portable infrared camera. The heating behavior of BP/gelatin/carrageenan phase‐change gel was photographed under the microscope.

### Preparation of Prolactin‐Loaded Core–Shell Microneedles

About 200 µL of a 10% PLGA acetone solution was taken, and it was centrifuged in a PDMS microneedle mold. Any excess PLGA solution was removed and it was dried in a vacuum oven to form a PLGA film coating the interior surfaces of the microneedle cavities. A microneedle shell was filled with 100 µL of a mixed material containing prolactin, 20% gelatin, 0.65% carrageenan, and 0.2 mg mL^−1^ BP at 39.3 °C to form a phase‐change core material, and any excess phase‐change material from the backing layer was removed. After the phase‐change material cooled and solidified, 50 µL of a 10% PLGA solution was added to the mold, and the solvent was centrifuged and evaporated to shape a cap layer on the bottom of the microneedles. Finally, a 20% PVA solution was added to the mold to form a soluble‐backing layer. The surface morphology of the prepared core–shell microneedle array was observed under an optical microscope and a scanning electron microscope.

### Mouse Ex Vivo Skin Microneedle Insertion Test

After insertion of microneedles into the excised mouse skin, the tissue sections were subsequently fixed and stained with HE. Then the puncture sites were examined on the skin using a light microscope.

### Mechanical Strength Test

Core–shell microneedles loaded with prolactin were positioned horizontally on a fixed station within the displacement–force testing apparatus, ensuring their tips faced upward. The force sensor was securely mounted above the stationary platform and approached the microneedles at a controlled velocity of 0.2 mm s^−1^. The measurement commenced upon initial contact between the sensor and the microneedle tips, concluding after the sensor had advanced 0.9 mm.

### Photothermal Conversion Test of Microneedles

In the photothermal conversion test, a microneedle was subjected to NIR irradiation with a 1.5 W cm^−2^ power density and 808 nm wavelength. Temperature variations and corresponding thermal images were recorded utilizing a thermal imaging device. During the cyclical on/off phases of microneedle photothermal conversion, NIR radiation continued until the temperature of microneedle reached a predefined threshold. Subsequently, when the temperature decreased to room temperature, the subsequent cycle commenced. In the NIR‐triggered heating test on mouse skin, microneedles were applied to the dorsal skin of mice and exposed to NIR radiation at 1.5 W cm^−2^ to record temperature variations and thermal images.

### Determination of Water Content and Mass Loss

PLGA microneedle shells were incubated in PBS (pH = 7.4, 37 °C, and 70 rpm). At the end of the designated time points, the incubations were terminated by removing the PBS and rinsing with distilled water. The shells were then dried in a vacuum drying oven. The water content of the PLGA microneedle shells (*W*(*t*)) at time *t* was determined during the incubation period using the following formula: *W*(*t*) (%) = [(*W^t^*_wet_ – *W^t^*_dry_)/*W^t^*_wet_] × 100, where *W*_wet_ and *W*_dry_ represent the wet and dry weights of the PLGA microneedle shells, respectively. The mass loss percentage was counted as follows: mass loss (%) = [(*W*_0_ – *W^t^*_dry_)/*W*_0_] × 100, where *W*_0_ represents the microneedle shells’ initial weight.

### In Vitro Release Experiment

To elucidate the underlying mechanisms of drug release, FITC–BSA‐loaded photothermal‐responsive microneedles were categorized into two groups: one exposed to NIR for 2 min h^−1^ and the other without NIR exposure. These microneedles were then inserted into agar blocks, and the green fluorescence of FITC within the microneedles was observed hourly using a fluorescence microscope. For quantitative analysis of the drug release process, 1 µg of FITC–BSA ‐loaded photothermal‐responsive microneedles was similarly divided and treated. The samples were placed in a PBS solution and agitated in a shaker at 300 rpm at 37 °C. Each NIR irradiation cycle was for 1 h, during which samples were collected and quantified using a fluorescence analyzer at hourly intervals.

### In Vitro Biocompatibility Evaluation

NIH 3T3 cells were partitioned into five distinct groups and cultured in a 96‐well plate. Each group had five replicates, with an initial cell count of ≈4 × 10^3^ per well. The first group functioned as the blank control, while the second group was administered PLGA microneedle shells. The third group received BP/gelatin/carrageenan microneedle core phase‐change material treatment. The fourth group was exposed to microneedle backing material PVA, and the fifth group underwent combined microneedle and NIR treatment. After 24 h, cell viability was assessed utilizing CCK‐8 and confirmed with the Calcein‐AM/PI double stain kit.

### Hemolysis Assays

The prepared microneedles were dissolved in PBS and diluted to various concentrations. Mouse blood was centrifuged to remove the supernatant plasma, and then washed three times with PBS (3000 rpm, 5 min). The leach liquor of microneedles was combined with a 5% red blood cell solution and incubated for 2 h (37 °C). The optical density (OD) of the mixture was determined at 540 nm. Calculation of the hemolysis rate formula was as follows

(1) $$\begin{array}{cc} & \mathrm{Hemolysis}\;\mathrm{rate}\left(\%\right)\\ & \;=\left[\left(OD_{microneedles}-OD_{\mathrm{PBS}}\right)/\left(OD_{\mathrm{Ultrapurewater}}-OD_{\mathrm{PBS}}\right)\times 100\right] \end{array}$$

### Exploration of FFA Modeling Concentration in AML‐12 Cells

A 50 mm solution of palmitic acid (PA) and a separate 50 mm solution of oleic acid (OA) were prepared. These solutions were subsequently diluted to their final working concentrations using a 10% BSA solution. To investigate the modeling concentration of FFA in AML‐12 cells, they were divided into five distinct groups and cultivated them in a 96‐well plate. Each group was consisted of five replicates, and the initial cell count per well was ≈5 × 10^3^. After allowing 12 h for cell adhesion and growth, the first group was exposed to 10% BSA, while the FFA concentrations for groups 2–6 were set at 100, 200, 300, 400, and 500 µm, individually. Cell viability was evaluated post a 24 h treatment interval utilizing the CCK‐8 assay and Calcein/PI assay.

### Exploring the Impact of Prolactin Administration on Cell Viability

AML‐12 cells were segregated into six groups and subsequently cultured in a 96‐well microtiter plate, wherein each group was consisted of five replicates. The initial cell count per well was ≈5 × 10^3^. Following 12 h of cell wall‐attached growth, one group was acted as the control, and other six groups were exposed to prolactin concentrations of 1, 10, 20, 50, 100, and 500 µg L^−1^, respectively. Cell viability was evaluated following a 24 h treatment period using the CCK‐8 assay kit.

### Exploring the Optimal Concentrations for Administering Prolactin In Vitro

To investigate the impact of various concentrations of prolactin on inhibiting lipid accumulation in normal mouse liver cells (AML‐12), the cells were categorized into six distinct groups and subsequently cultured in a 24‐well microtiter plate. Each group consisted of three replicates, with an initial cell count of ≈2.5 × 10^5^ per well. Following a 12 h period for cell adhesion and growth, the first control group was treated with 10% BSA, while groups 2–7 were exposed to prolactin concentrations of 1, 10, 20, 50, 100, and 500 µg L^−1^, respectively. Concurrently, groups 2–7 were also treated with a 200 µm FFA. After a 24 h incubation period, cells from each group were harvested. Triglyceride levels in the cells were determined using the triglyceride detection kit (Pulilai, China), while protein levels in the samples were determined using the BCA protein quantification kit. Both measurements were performed using an ELISA reader. Additionally, a semiquantitative assessment of lipid accumulation in each group was conducted using the Oil Red O staining method.

### Determination of Secondary Structure of Prolactin

CD spectropolarimetry (Jasco J‐815) was used to characterize any deviations in prolactin's secondary structure. Prolactin stability, preserved after the patch fabrication and subsequent release process, was measured. Samples were performed at 1 nm intervals between 190 and 260 nm for far‐UV measurements. Sample spectra were compared with the spectra of fresh prolactin solution.

### In Vitro Efficacy Evaluation

To assess the efficacy of each treatment in vitro, AML‐12 cells were categorized into five distinct groups, with one group receiving treatment solely from blank microneedles. The second group received a combination of 200 µm FFA and blank microneedles. In the third group, cells were treated with blank microneedles, 200 µm FFA, and 100 µg L^−1^ prolactin. The fourth group was exposed to 200 µm FFA modeling followed by treatment with microneedles containing 100 µg L^−1^ prolactin. Finally, the fifth group underwent treatment with both 100 µg L^−1^ prolactin microneedles and NIR after being modeled with 200 µm FFA. To quantitatively measure triglyceride levels in each cellular group, a triglyceride quantitative detection kit was utilized. Additionally, lipid accumulation in each group was visualized using the Oil Red O and BODIPY staining method.

### Exploring the Mechanism of Prolactin in Inhibiting Lipid Accumulation in Liver Cells In Vitro

qRT‐PCR was used to assess the transcriptional levels of the gene encoding *Prlr*, *Cd36*, *Srebp1c*, *Acc1*, *Pparα*, and *Cpt1a* in the liver cells of each treatment group. In brief, after total RNA was extracted, followed by reverse transcription employing the PrimeScript RT kit. qRT‐PCR was conducted utilizing an SYBR Green‐based RT‐PCR master mix. WB was utilized to detect the protein expression of PRLR, CD36, p‐STAT‐5, and STAT‐5 in the liver cells of each treatment group. WB analyses involved the extraction of proteins from both tissue and cellular samples. Subsequently, protein concentrations were determined, and lysates totaling 40 µg were subjected to electrophoresis. The proteins were subsequently transferred to polyvinylidene difluoride membranes, which were then sequentially incubated with primary and secondary antibodies.

### In Vivo Animal Experiments

The mice were allocated to five distinct groups at random. The first group functioned as the normal diet control and received blank microneedling treatment along with a subcutaneous injection of PBS solution. The second group was the HFD model group, which also received blank microneedle treatment and a subcutaneous injection of PBS solution. The third group was administered prolactin subcutaneously and received blank microneedling treatment during high‐fat feeding. The fourth group received prolactin through microneedles and a subcutaneous injection of PBS solution during high‐fat feeding. The fifth group received prolactin through microneedles combined with NIR irradiation and a subcutaneous injection of PBS solution during high‐fat feeding.

The dosage for prolactin administration was primarily determined based on the previous study findings.^[^ ^17^ ^]^ In mice subjected to a high‐fat diet, the levels of free fatty acids increased with the duration of high‐fat modeling. This increase in free fatty acids levels inhibits the secretion of pituitary prolactin. Consequently, a strategy that involved increasing both the frequency and dose of administration as the modeling time extended was adopted, aiming to enhance circulating prolactin levels. Treatments were administered once a week for the first 5 weeks with a prolactin of 40 µg kg^−1^, once 3 days from weeks 6 to 8 with a prolactin of 60 µg kg^−1^, and daily in the 9–10 weeks with a prolactin of 80 µg kg^−1^. The final dosage was determined based on pre‐experimental results and previous research.^[^ ^54^, ^61^ ^]^ The specific time points for prolactin administration was at ZT10 (6 p.m.).

Blood samples were obtained via the tail veins of mice at ZT0, ZT4, ZT8, ZT12, ZT16, and ZT20 on the following day before euthanizing the mice at the end of the 10th week of modeling to measure prolactin levels. The levels of prolactin were quantified utilizing ELISA kits, adhering strictly to the protocols provided by the manufacturer. Tissue samples from the liver were collected during euthanasia to assess both the liver's weight and triglyceride levels. HE staining, along with Oil Red O staining, was employed to examine mouse livers steatosis. Skin, heart, spleen, lungs, and kidneys were collected from mice for HE staining to observe pathological changes and evaluate drug administration safety. qRT‐PCR was employed to quantify gene levels of *Prlr*, *Cd36*, *Srebp1c*, *Acc1*, *Pparα*, and *Cpt1a* in liver tissue from each treatment group. WB was employed to assess the protein expression of PRLR, CD36, p‐STAT‐5, and STAT‐5 in liver cells from each treatment group.

### Histological Procedures

For HE staining, first, fresh liver specimens were fixed in 4% paraformaldehyde (≥24 h). Then the samples were dehydration through a graded series of alcohol solutions at concentrations of 70%, 80%, and 90%, followed by paraffin embedding. Sections of 4 µm were stained using a standard HE alcoholic procedure. Sections of 8 µm were stained using Oil Red O staining. The same experimental procedure was replicated using AML‐12 cells.

### Statistical Analysis

The results in this study were analyzed by the software GraphPad Prism 8.0. The statistical analysis was evaluated using a two‐sided Student's *t*‐test or an analysis of variance (ANOVA). *P < 0.05, **P < 0.01, ***P < 0.001, n.s., not significant difference.

## Conflict of Interest

The authors declare no conflict of interest.

## Author Contributions

H.Y., W.T., and D.H. contributed equally to this work. Y.B., Y.Z., and P.Z. conceived the research idea and designed the experimental procedures. H.Y. and W.T. conducted the experiments and performed data analysis. H.Y. was responsible for organizing the content, authoring the manuscript, and arranging the figures. D.H. reviewed the grammar and figures. J.J.G. guided the experimental operations. Finally, Y.Z. revised the manuscript.

## Supporting information



Supporting Information

## Data Availability

The data that support the findings of this study are available from the corresponding author upon reasonable request.
