# Supplementary material for: Bioinspired Prolactin Pulse Release from Responsive Microneedles for Inhibiting Fatty Liver Formation
Source: Adv Sci (Weinh). 2025 Jun 30;12(36):e08364. doi: 10.1002/advs.202508364 (PMC12463070; doi:10.1002/advs.202508364)
Supplement: Supplementary file 1 — Supporting Information [file ADVS-12-e08364-s001.docx]

Supporting Information

**Bioinspired Prolactin Pulse Release from Responsive Microneedles for Inhibiting Fatty Liver Formation**

*Hongli Yin, Wenjuan Tang, Danqing Huang, JingJing Gan, Pengzi Zhang*, Yuanjin Zhao*, Yan Bi**

**Figures**

Figure S1. The investigation into the mixing ratios of gelatin and carrageenan.

Figure S2. Evaluation of photothermal response characteristics of Gelatin/Carrageenan/BP Mixtures.

Figure S3. The scanning electron microscope images of microneedles.

Figure S4. Stability investigation of PLGA microneedle shell.

Figure S5. The observation of the FITC–BSA release from the microneedles.

Figure S6. Biocompatibility and safety evaluation of microneedles.

Figure S7. The effects of varying concentrations of FFA on AML-12 cells.

Figure S8. The impact of differing prolactin concentrations on the viability and lipid accumulation in AML-12 cells.

Figure S9. Circular dichroism spectra of treated prolactin and fresh prepared prolactin.

Figure S10. The expression of genes implicated in lipid metabolism and synthesis in different in vitro treatment groups.

Figure S11. The heating characteristics of prolactin-loaded responsive microneedles under NIR irradiation.

Figure S12. The expression of genes implicated in lipid metabolism and synthesis in different in vivo treatment groups.

Figure S13. The protein levels of PRLR, pSTAT5, STAT5, and CD36 in different in vivo treatment groups.

Figure S14. Biosafety evaluation of microneedles by the HE staining.

**Tables**

Table S1. The serum prolactin levels of each group mice changed every 4 hours within one day before the mouse was euthanized.

Table S2. Primer sequences used in qRT-PCR assays.


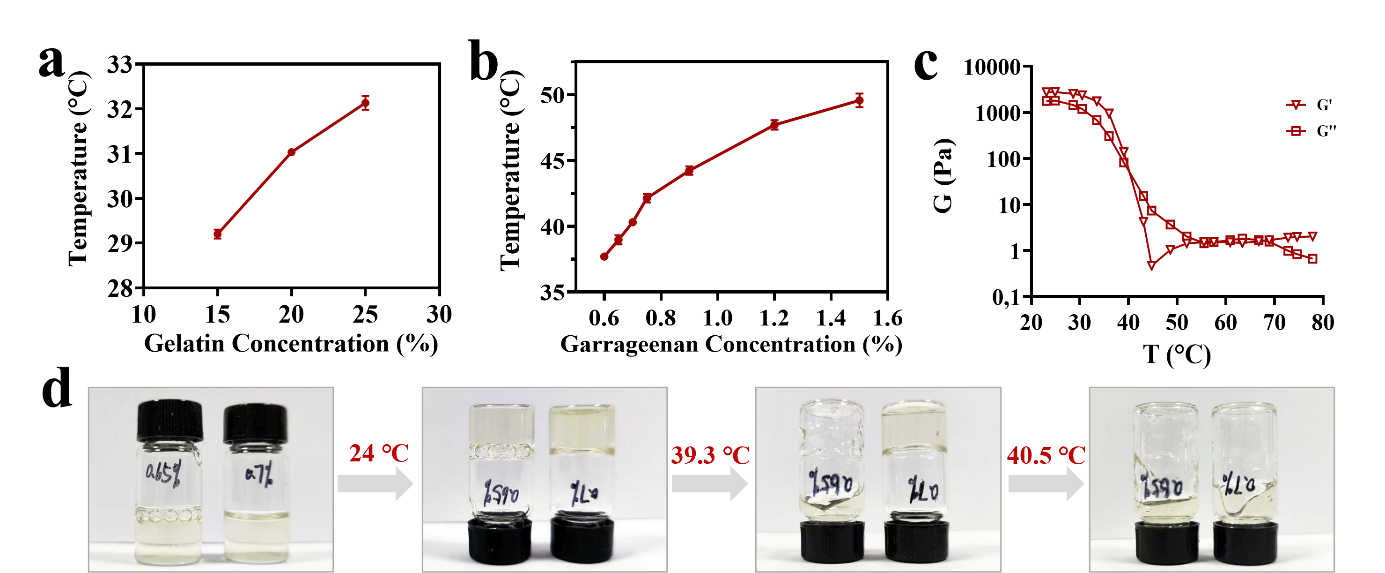


**Figure S1.** The investigation into the mixing ratios of gelatin and carrageenan. a) Influence of carrageenan concentrations on the critical temperature (*n* = 3). b) Influence of gelatin concentrations on the critical temperature (*n* = 3). c) The critical temperature of 20% gelatin and 0.65% carrageenan mixture. d) The states of gelatin mixed with different concentrations of carrageenan before and after heated at 39.3 ℃ and 40.5 ℃. a, b) Mean ± SD, *n* = 3.


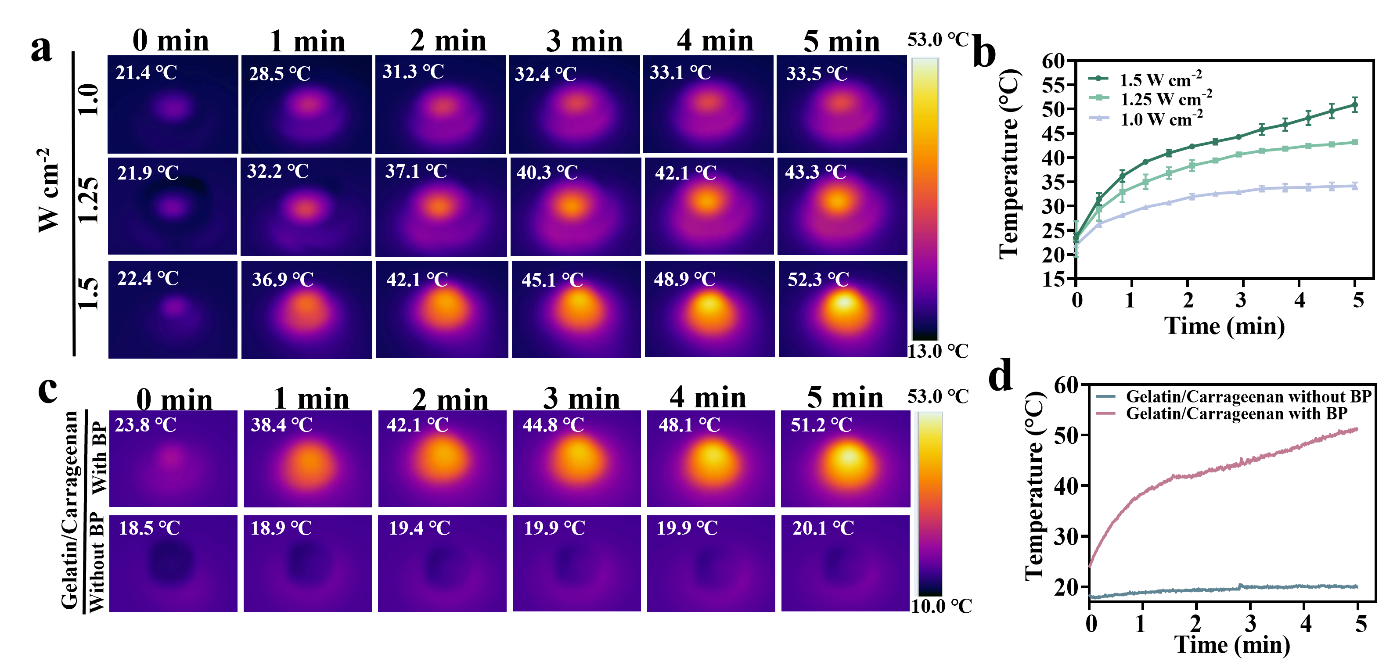


**Figure S2.** Evaluation of photothermal response characteristics of Gelatin/Carrageenan/BP Mixtures. a) Thermal images of microneedles core materials (20% gelatin + 0.65% carrageenan + 0.2 mg mL^-1^ BP) before and after different NIR power irradiation. b) The relationship between temperature of microneedles core materials and time under different NIR power (mean ± SD, *n* = 3). c) Thermal images of core materials containing and without BP microneedles before and after 1.5 W cm^-2^ NIR irradiation. d) Photothermal response heating curves without and with BP core materials.


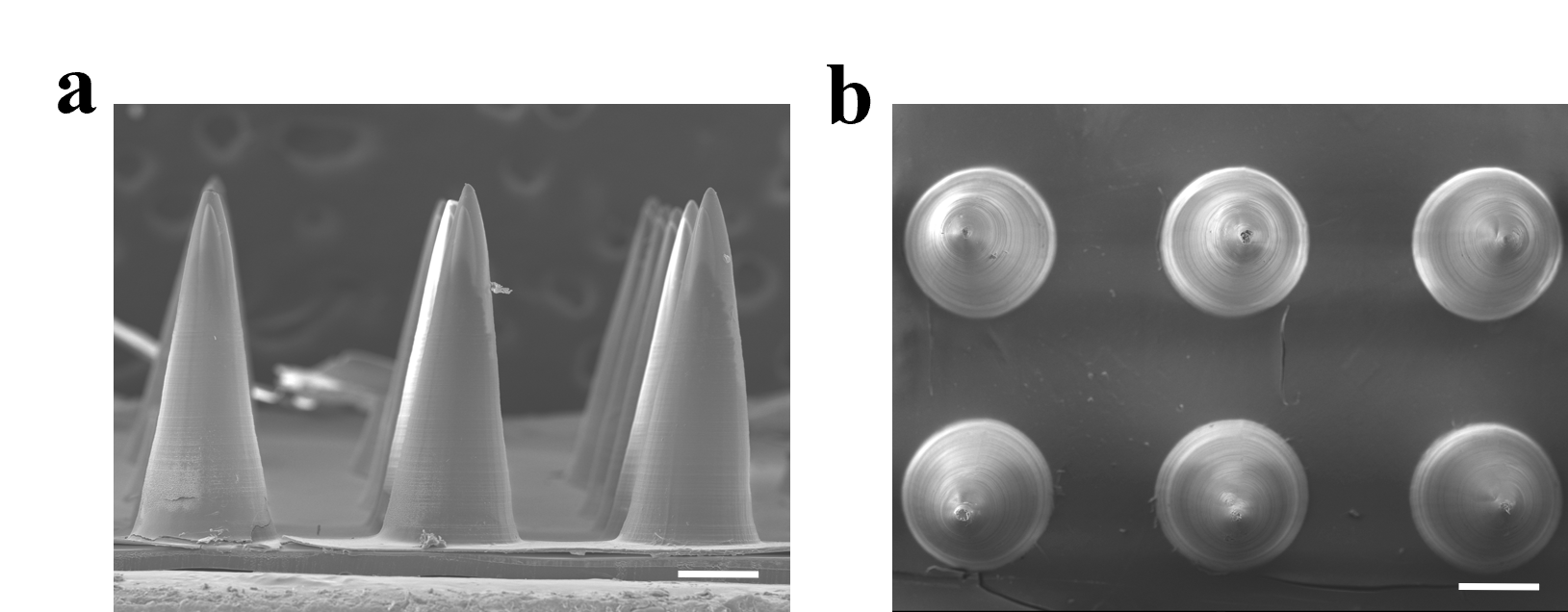


**Figure S3.** The scanning electron microscope images of microneedles. a) Side and b) top scanning electron microscope views of core-shell microneedles (scale bar = 200 μm).


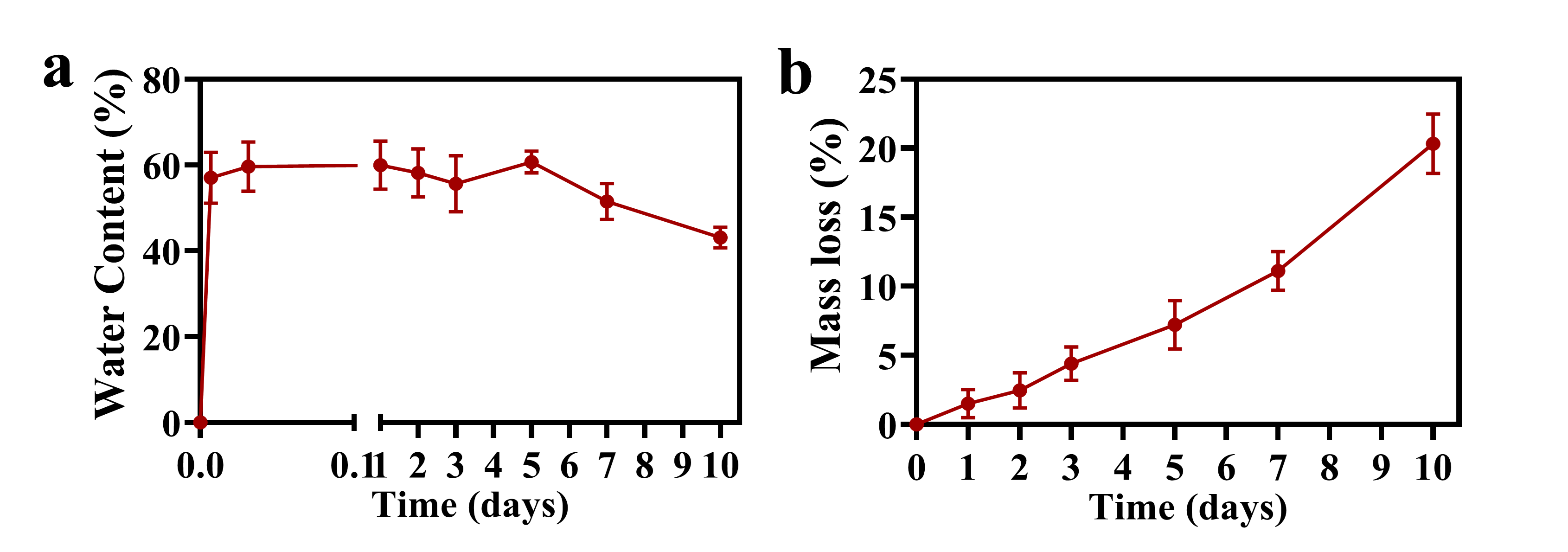


**Figure S4**. Stability investigation of PLGA microneedle shell. a) The kinetics of water content and b) the mass loss of PLGA microneedle shell. (mean ± SD, *n* = 5)


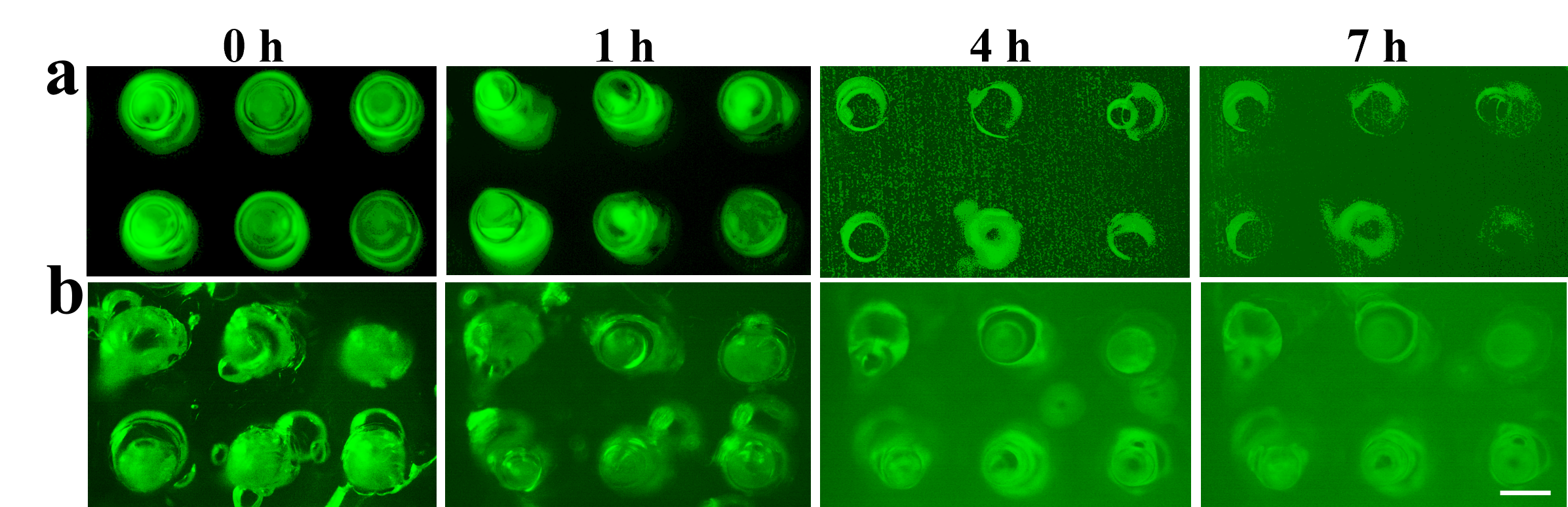


**Figure S5.** The observation of the FITC–BSA release from the microneedles. a) After 0, 1, 4, and 7 cycles of NIR irradiation (with one hour interval between each irradiation) and b) after 0, 1, 4, and 7 h of without NIR irradiation, fluorescence photos of loading FITC–BSA microneedles in agar blocks (scale bar = 300 μm).


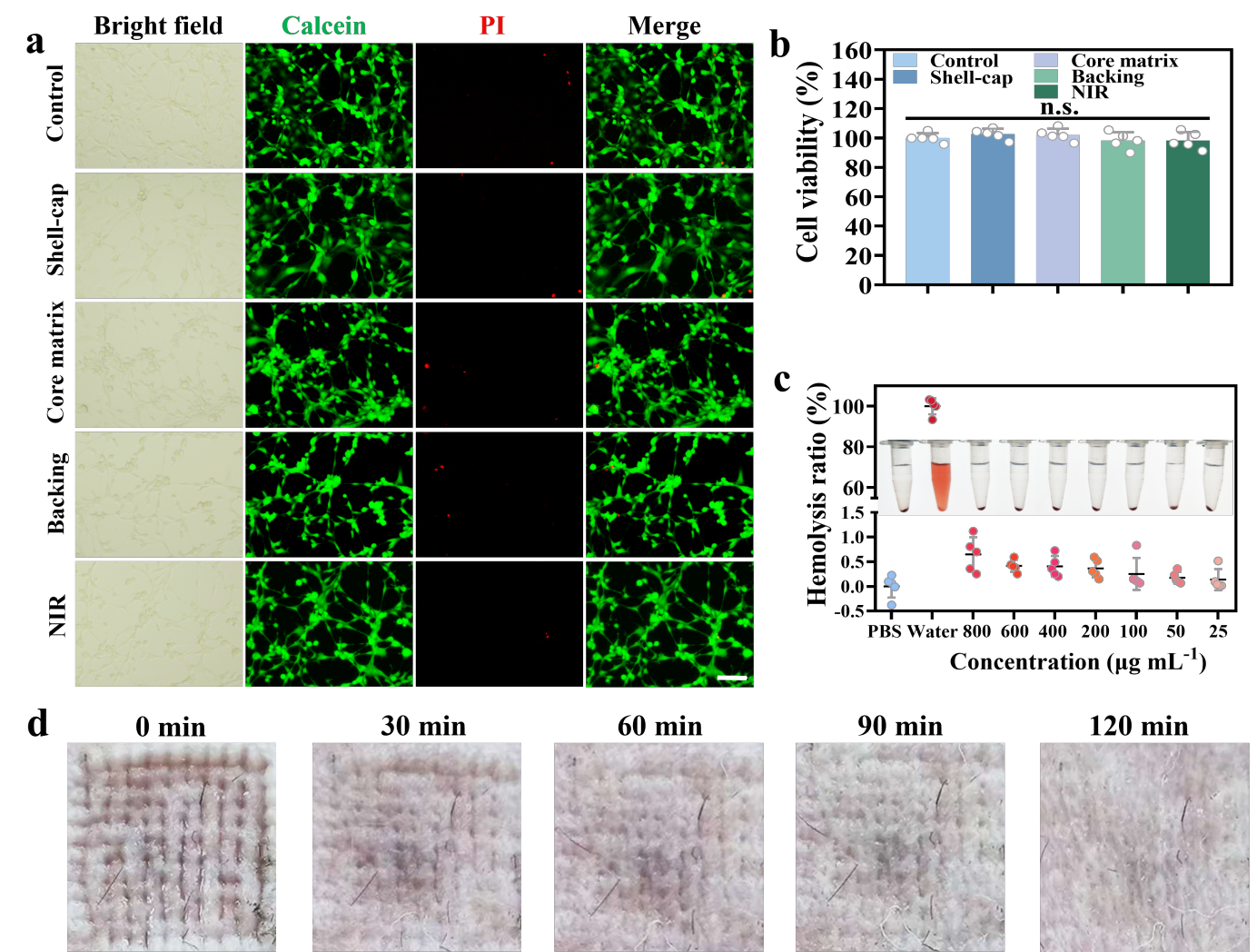


**Figure S6.** Biocompatibility and safety evaluation of microneedles. a) Calcein/PI assay, with images featuring 100 μm scale bar, and b) CCK-8 assay were utilized to evaluate the viability and mortality of 3T3 cells. c) The hemolysis rates of red blood cells after incubation with varying concentrations of microneedle extracts. d) The insertion efficiency of microneedles and the recovery of the skin at the site of penetration after microneedles into mouse skin. b, c) Mean ± SD, *n* = 5.


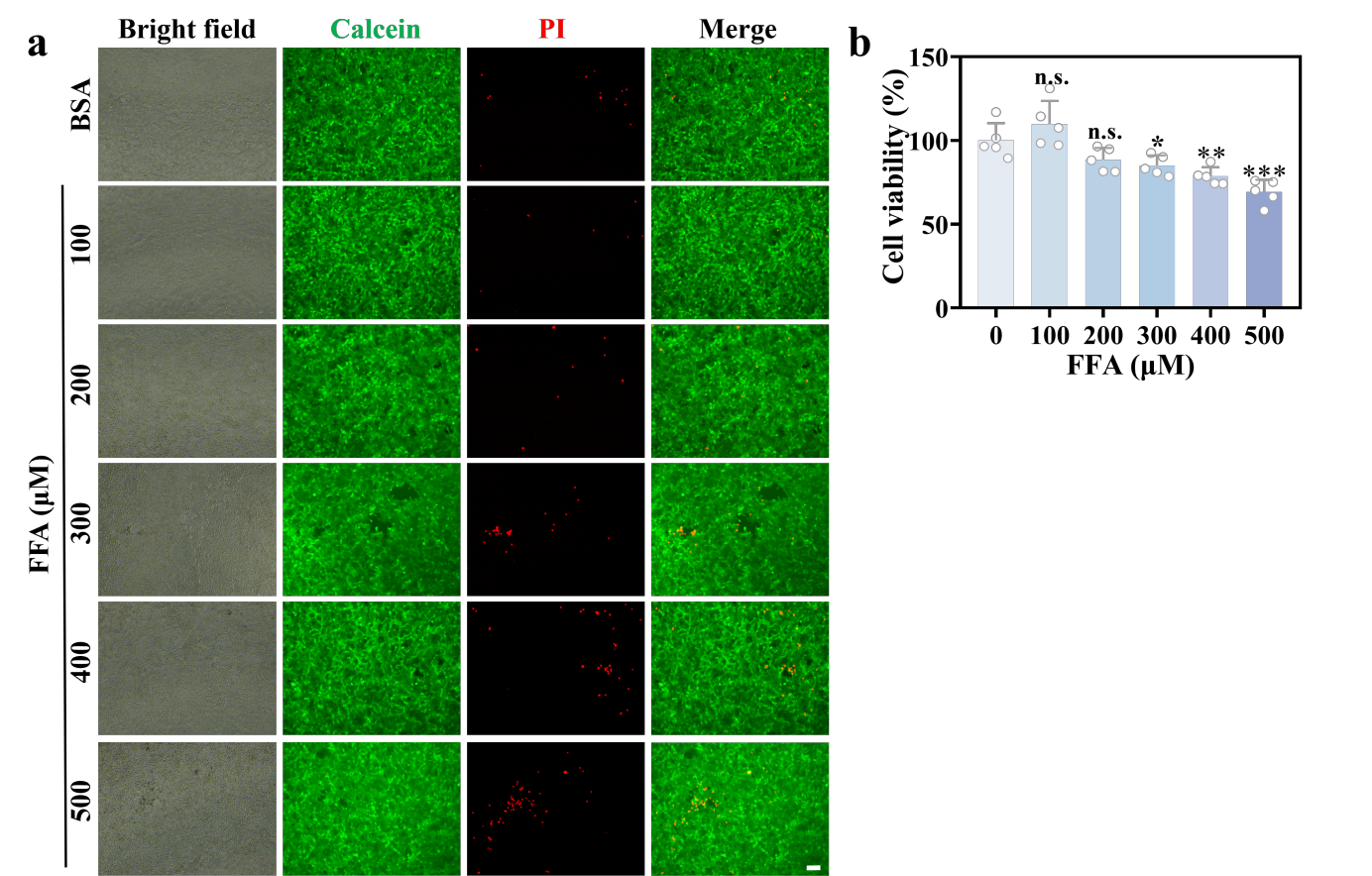


**Figure S7.** The effects of varying concentrations of FFA on AML-12 cells. a) Calcein/PI method (scale bar = 100 μm) and b) CCK-8 method (mean ± SD, *n* = 5) were used to evaluate the viability and mortality of AML-12 cells.


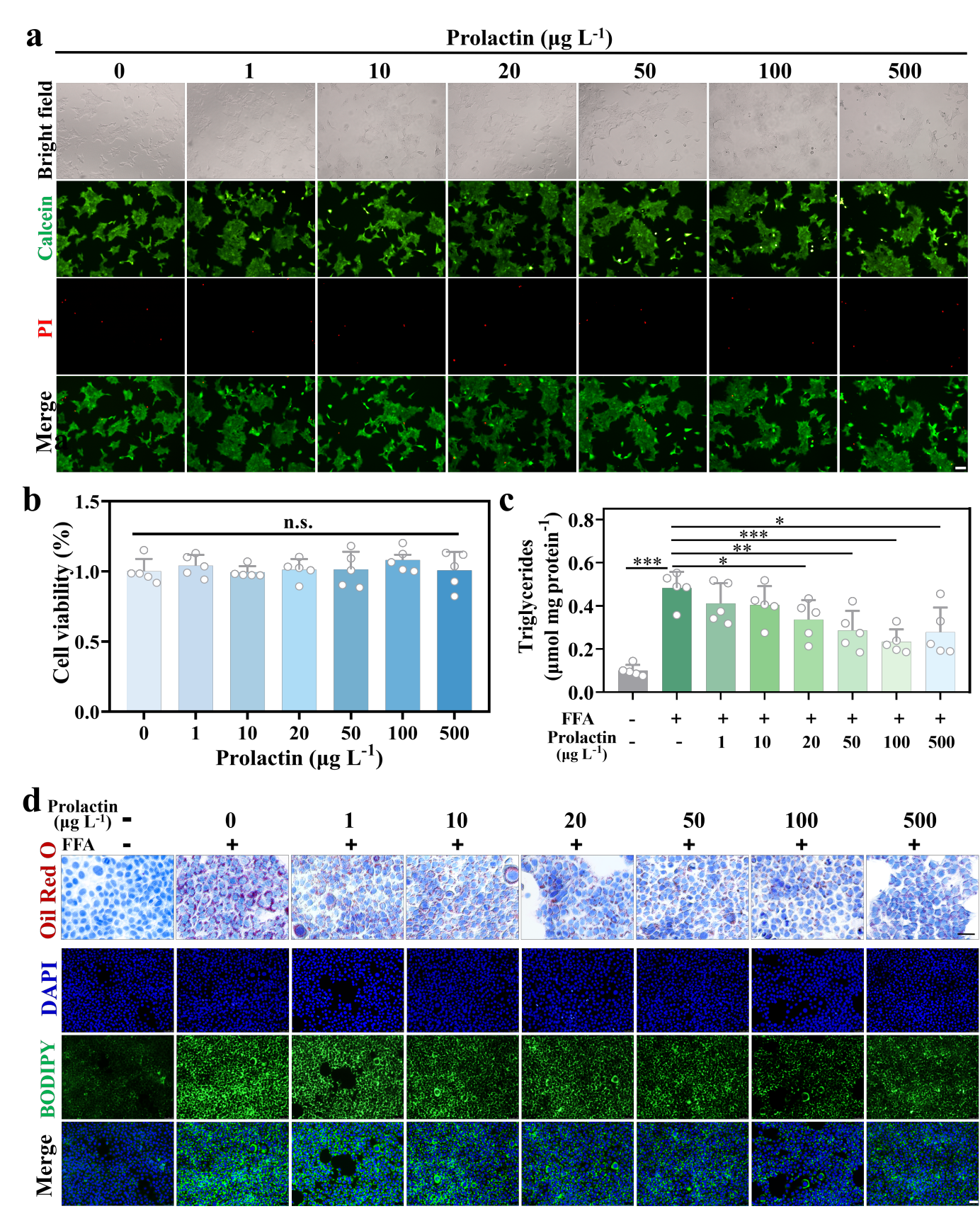


**Figure S8.** The impact of differing prolactin concentrations on the viability and lipid accumulation in AML-12 cells. a) Calcein/PI method (scale bar = 100 μm) and b) the CCK-8 assay were used to assess the effects of varying prolactin concentrations on the viability and mortality of AML-12 cells. c) The triglyceride levels of different concentrations of prolactin on AML-12 cells. d) Oil Red O and BODIPY staining were used to assess the lipid-lowering effect of each different prolactin concentrations (scale bar = 50 μm). b, c) Mean ± SD, *n* = 5.


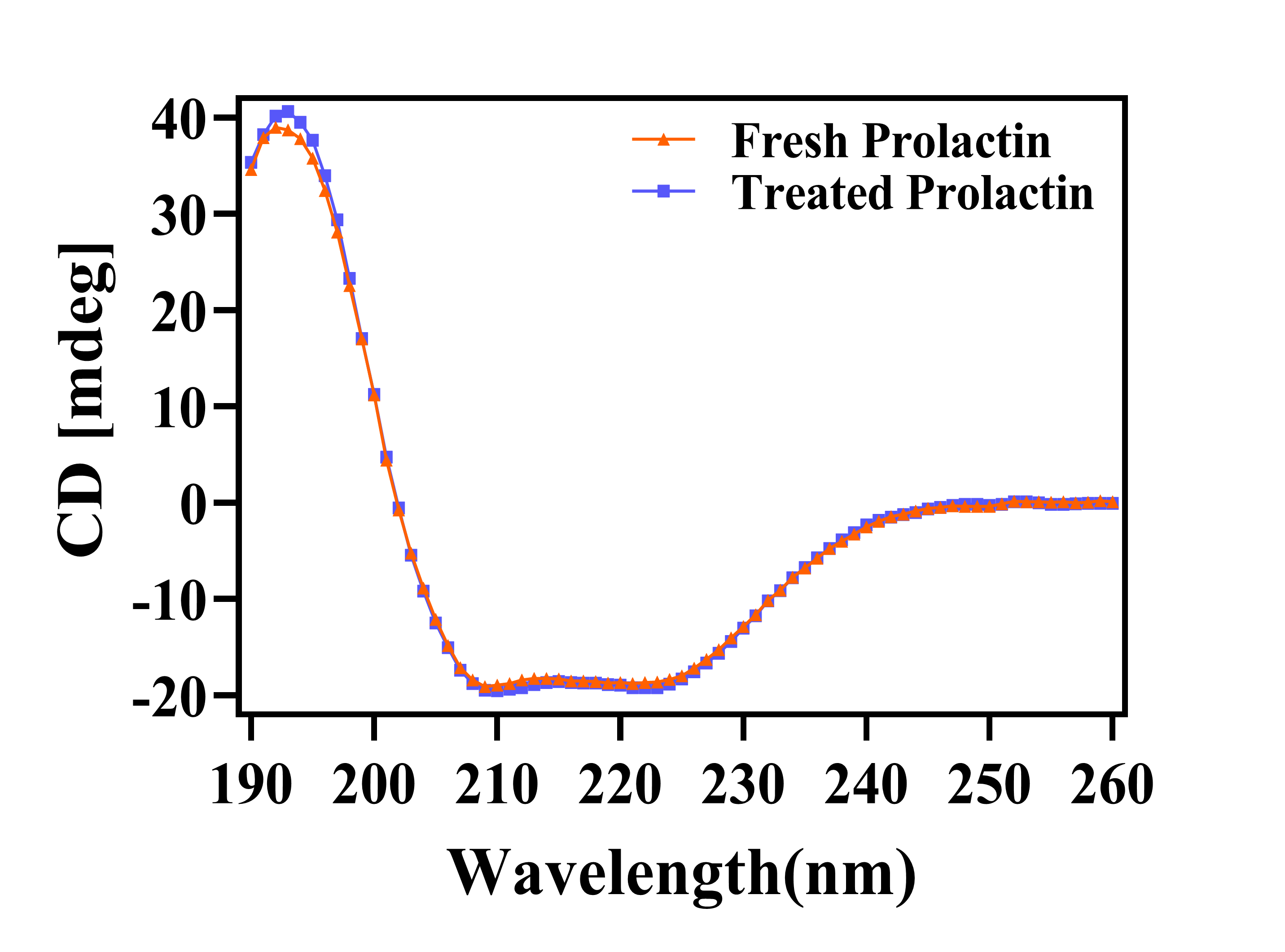


**Figure S9.** Circular dichroism spectra of treated prolactin and fresh prepared prolactin.


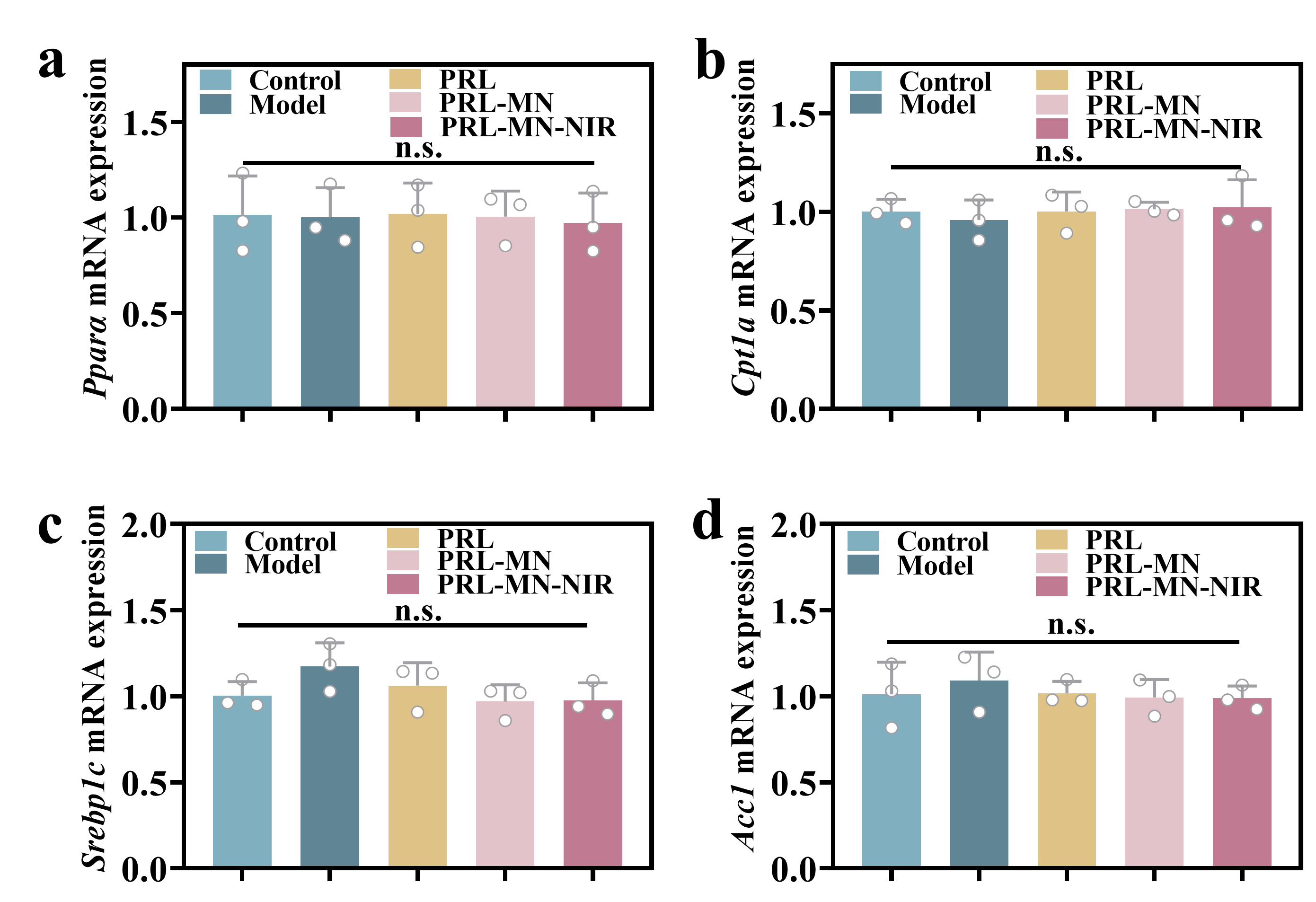


**Figure S10.** The expression of genes implicated in lipid metabolism and synthesis in different in vitro treatment groups. The a) *Pparα*, b) *Cpt1a*, c) *Srebp1c* and d) *Acc1* mRNA expression levels in each treatment group. (mean ± SD, *n* = 3)


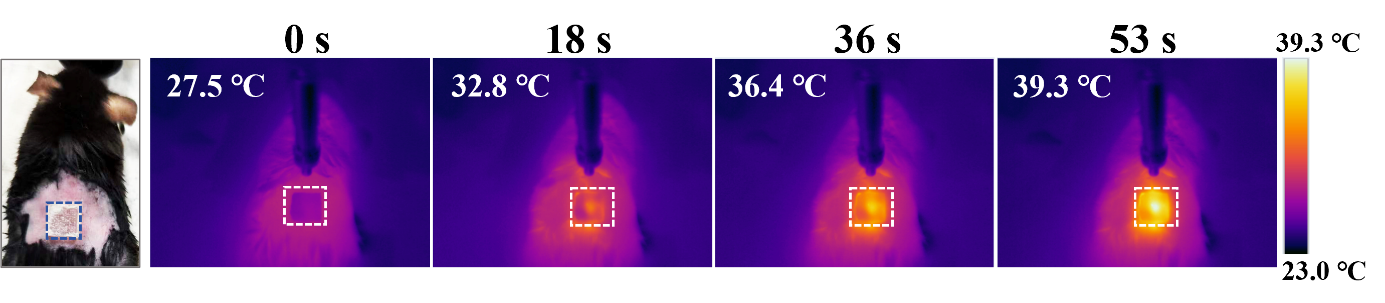


**Figure S11.** The heating characteristics of prolactin-loaded responsive microneedles under NIR irradiation.


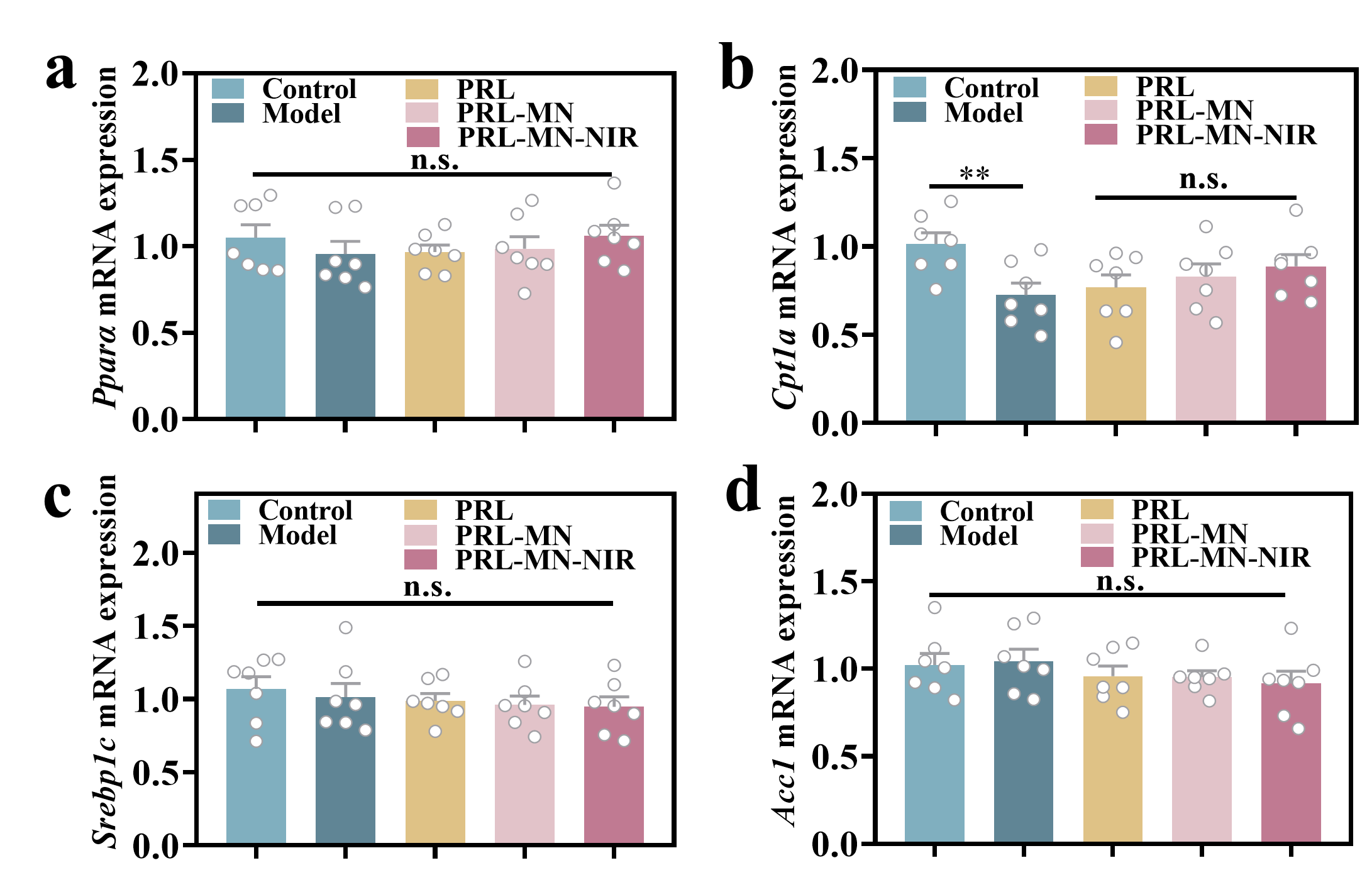


**Figure S12.** The expression of genes implicated in lipid metabolism and synthesis in different in vivo treatment groups. mRNA levels of a) *Pparα*, b) *Cpt1a*, c) *Srebp1c*, and d) *Acc1* were measured in each treatment group. (mean ± SEM, *n* = 7)


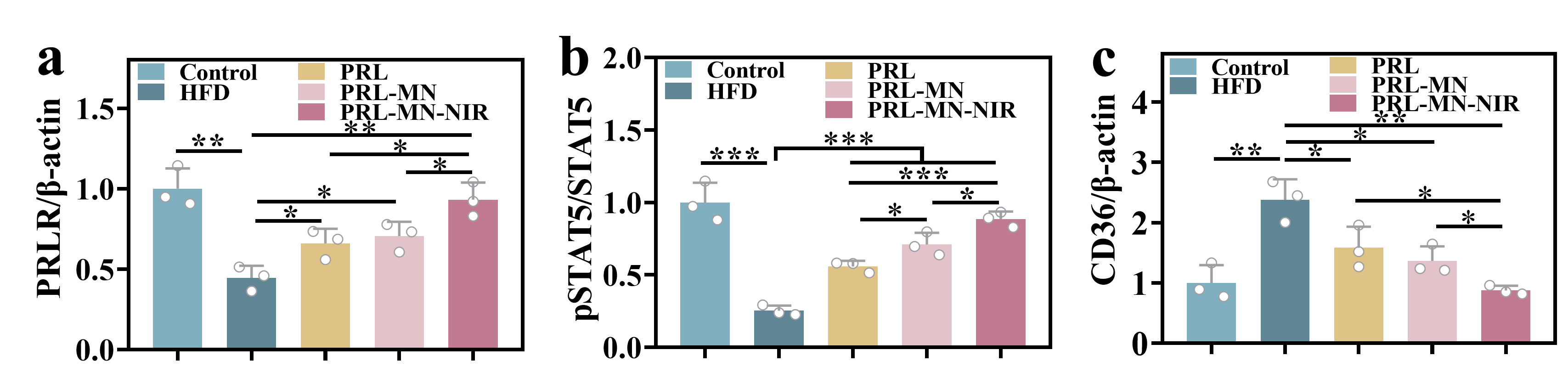


**Figure S13.** The protein levels of PRLR, pSTAT5, STAT5, and CD36 in different in vivo treatment groups. (mean ± SD, *n* = 3)

**
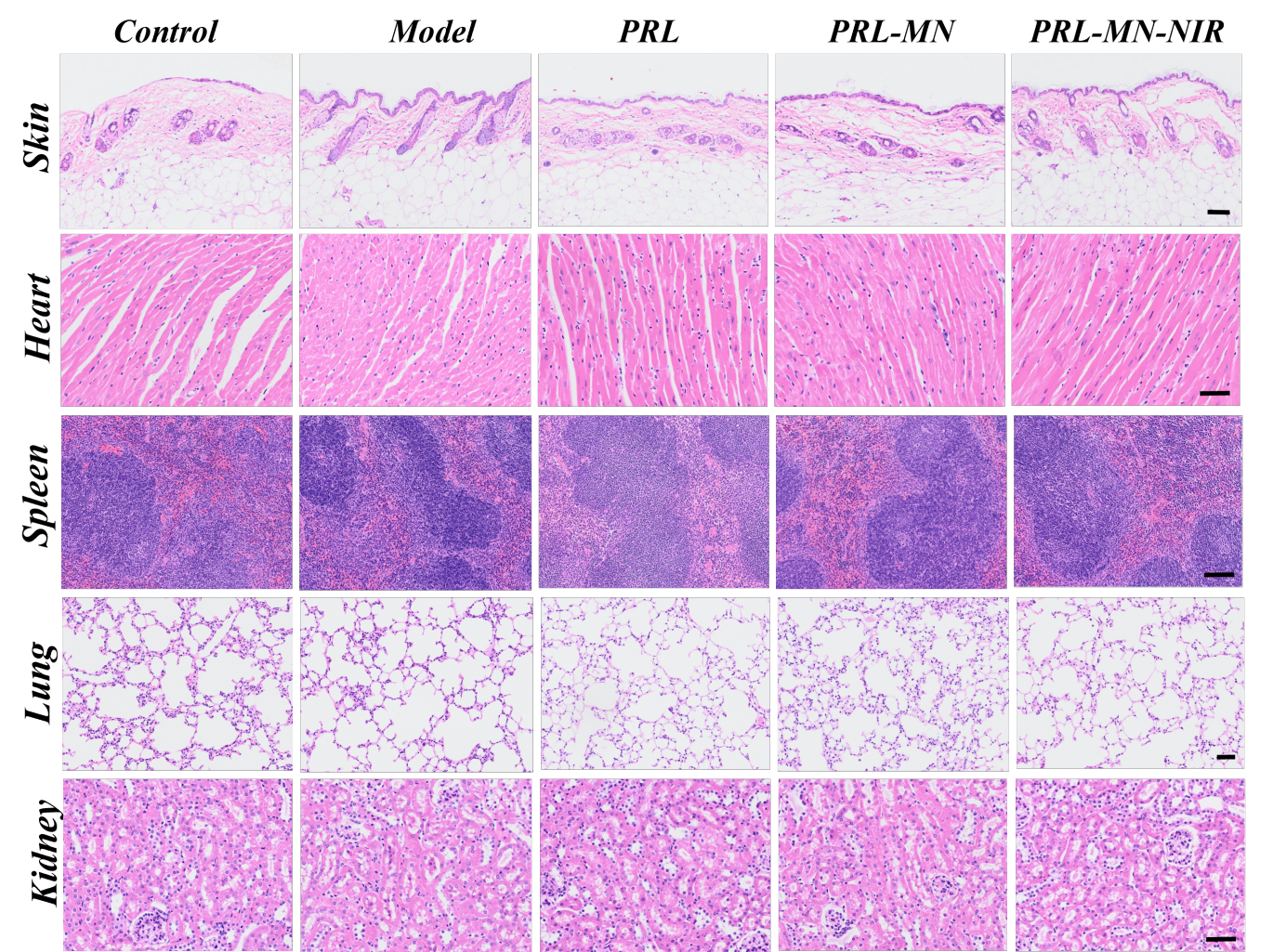
**

**Figure S14.** Biosafety evaluation of microneedles by the HE staining. The organs include skin, heart, lung, kidney, with images featuring 50 µm scale bars respectively, and spleen, with images featuring 100 µm scale bars.

**Table S1.** The serum prolactin levels of each group mice changed every 4 hours within one day before the mouse was euthanized. (mean ± SD, *n* = 7, μg L^-1^)

| **Time** | **Control** | **Model** | **PRL** | **PRL-MN** | **PRL-MN-NIR** |
| --- | --- | --- | --- | --- | --- |
| ZT0 (8 a.m.) | 16.81 ± 2.72 | 13.12 ± 3.24**^#^** | 14.76 ± 3.71 | 18.56 ± 4.55* | 18.94 ± 4.91* |
| ZT4 (12 p.m.) | 22.94 ± 4.19 | 17.30 ± 4.08**^#^** | 20.58 ± 4.38 | 23.91 ± 7.00 | 23.85 ± 6.75* |
| ZT8 (16 p.m.) | 33.38 ± 8.56 | 22.33 ± 6.50**^#^** | 28.47 ± 10.28 | 31.35 ± 9.01 | 31.57 ± 8.10* |
| ZT12 (20 p.m.) | 50.06 ± 11.13 | 28.60 ± 7.55**^##^** | 49.51 ± 10.24*** | 37.53 ± 7.03* | 45.63 ± 6.62***, **^@^** |
| ZT16 (24 a.m.) | 30.79 ± 7.47 | 21.59 ± 6.09**^#^** | 25.71 ± 5.17 | 31.77 ± 6.72* | 32.54 ± 6.19**, **^&^** |
| ZT20 (4 a.m.) | 26.37 ± 5.03 | 19.19 ± 5.16**^#^** | 23.70 ± 8.53 | 25.90 ± 6.92 | 25.77 ± 5.52* |

*vs* control ^#^*P*<0.05, ^##^*P*<0.01; *vs* model **P*<0.05, ***P*<0.01, ****P*<0.001; *vs* PRL ^&^*P*<0.05; *vs* PRL-MN ^@^*P*<0.05

**Table S2.** Primer sequences used in qRT-PCR assays.

| **Species** | **Name** | **Sequence (5’→3’)** |
| --- | --- | --- |
| Mouse | *Prlr*F | GAGAAGGGCAAGTCTGAAGAAC |
| Mouse | *Prlr*R | GGGATGGCATTAGCCGCTC |
| Mouse | *Cd36*F | ATGGGCTGTGATCGGAACTG |
| Mouse | *Cd36*R | GTCTTCCCAATAAGCATGTCTCC |
| Mouse | *Srebp1c*F | GATGTGCGAACTGGACACAG |
| Mouse | *Srebp1c*R | CATAGGGGGCGTCAAACAG |
| Mouse | *Acc1*F | ATGGGCGGAATGGTCTCTTTC |
| Mouse | *Acc1*R | TGGGGACCTTGTCTTCATCAT |
| Mouse | *Ppar-a*F | AGAGCCCCATCTGTCCTCTC |
| Mouse | *Ppar-a*R | ACTGGTAGTCTGCAAAACCAAA |
| Mouse | *Cpt1a*F | CTCCGCCTGAGCCATGAAG |
| Mouse | *Cpt1a*R | CACCAGTGATGATGCCATTCT |
| Mouse | *Beta-actin*F | GGCTGTATTCCCCTCCATCG |
| Mouse | *Beta-actin*R | CCAGTTGGTAACAATGCCATGT |
